# Synoptic sampling and principal components analysis to identify sources of water and metals to an acid mine drainage stream

**DOI:** 10.1007/s11356-017-9038-x

**Published:** 2017-06-06

**Authors:** Patrick Byrne, Robert L. Runkel, Katherine Walton-Day

**Affiliations:** 1 School of Natural Sciences and Psychology, Liverpool John Moores University, Liverpool, L3 3AF UK; 2 U. S. Geological Survey, Denver Federal Center, PO Box 25046, Mail Stop 415, Denver, CO 80225 USA

**Keywords:** Metals, Acid mine drainage, Tracer injection, Synoptic sampling, Principal components analysis, Minnesota Mine

## Abstract

Combining the synoptic mass balance approach with principal components analysis (PCA) can be an effective method for discretising the chemistry of inflows and source areas in watersheds where contamination is diffuse in nature and/or complicated by groundwater interactions. This paper presents a field-scale study in which synoptic sampling and PCA are employed in a mineralized watershed (Lion Creek, Colorado, USA) under low flow conditions to (i) quantify the impacts of mining activity on stream water quality; (ii) quantify the spatial pattern of constituent loading; and (iii) identify inflow sources most responsible for observed changes in stream chemistry and constituent loading. Several of the constituents investigated (Al, Cd, Cu, Fe, Mn, Zn) fail to meet chronic aquatic life standards along most of the study reach. The spatial pattern of constituent loading suggests four primary sources of contamination under low flow conditions. Three of these sources are associated with acidic (pH <3.1) seeps that enter along the left bank of Lion Creek. Investigation of inflow water (trace metal and major ion) chemistry using PCA suggests a hydraulic connection between many of the left bank inflows and mine water in the Minnesota Mine shaft located to the north-east of the river channel. In addition, water chemistry data during a rainfall-runoff event suggests the spatial pattern of constituent loading may be modified during rainfall due to dissolution of efflorescent salts or erosion of streamside tailings. These data point to the complexity of contaminant mobilisation processes and constituent loading in mining-affected watersheds but the combined synoptic sampling and PCA approach enables a conceptual model of contaminant dynamics to be developed to inform remediation.

**Electronic supplementary material:**

The online version of this article (doi:10.1007/s11356-017-9038-x) contains supplementary material, which is available to authorized users.

## Introduction

Contaminated drainage from historical and contemporary hard rock mining activities is recognised as one of the most pressing global water quality issues (Mayes et al. 2008; Palumbo-Roe et al. 2012; Hudson-Edwards 2016). Typically, contaminated drainage has multiple sources across a mineralised watershed and is often diffuse in nature (Byrne et al. 2013; Runkel et al. 2013). Consequently, effective remediation requires an accurate and detailed assessment of spatial patterns of contamination at the watershed-scale.

The synoptic mass balance approach for quantifying contaminant sources and loading has been used extensively within the USA as part of the Abandoned Mine Lands Initiative (Kimball et al. 2004; Kimball et al. 2007). Under steady-state flow conditions, stream and water inflow sites across the impacted watershed are sampled for constituents of interest. Constituent concentrations are combined with estimates of streamflow for each site to generate a spatial pattern of constituent loading for the watershed (Runkel et al. 2013). These spatially dense loading estimates can then be used to identify contaminant source areas and to prioritise remediation activities.

Sampling of water inflows is important to identify specific sources that account for any observed changes in stream chemistry and constituent loading. Patterns in the chemical characteristics of water inflows can be used to fingerprint the distinct geochemical signals of mined and unmined areas within a watershed. Principal components analysis (PCA) is a powerful statistical method for determining chemical similarity or distinction between spatially dense synoptic samples. PCA effectively rotates chemical data so as to visualise the greatest distinctions among groups of samples (Kimball et al. 2004). The large number of possible explanatory variables is reduced to a smaller number of principal components that represent a linear combination of the original variables. Despite some notable exceptions (Kimball et al. 2002; De Giudici et al. 2014), PCA has rarely been applied in synoptic sampling studies to characterise water inflows and to identify contaminant sources in mineralized catchments.

In this study, a combined synoptic sampling and principal components analysis approach is adopted in Lion Creek, a mineralized watershed receiving acid mine drainage in Colorado, USA. The objectives of this research are as follows: (i) to quantify the impacts of mining activity on stream water quality; (ii) to quantify the spatial pattern of constituent loading; and (iii) to identify inflow sources most responsible for observed changes in stream chemistry and constituent loading.

## Methodology

### Field setting

**Fig. 1:**
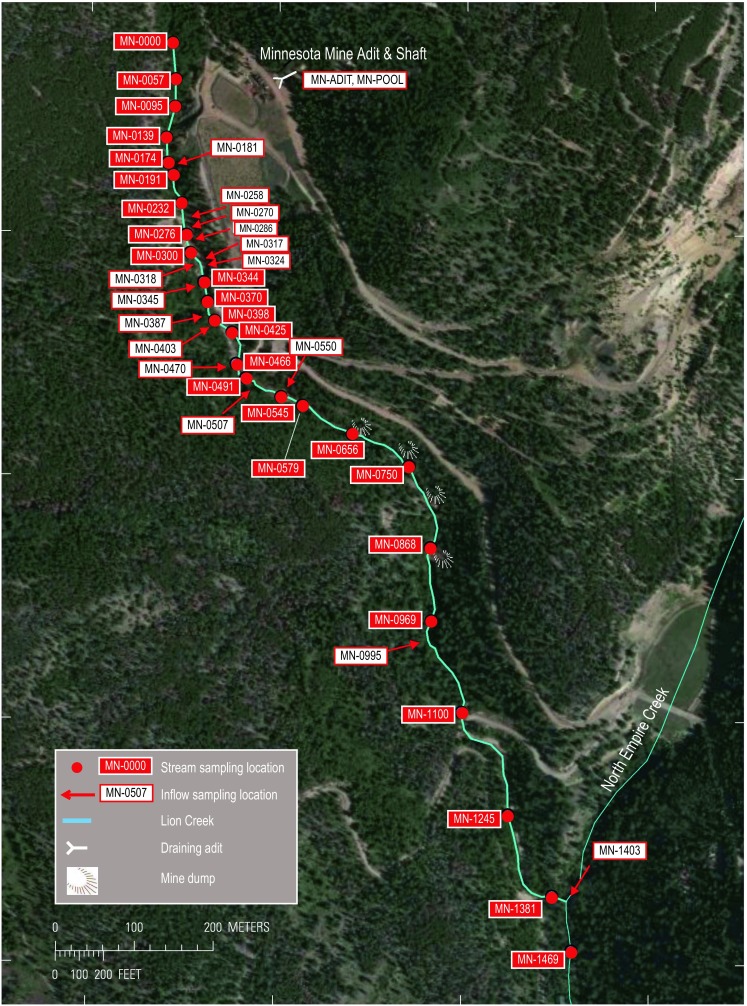
Location of Lion Creek showing stream and inflow samples. *Numbers associated with sample names* represent distance (in meters) below the tracer injection point (MN-0000). (Water samples were collected from a nested piezometer at MN-0324)

Lion Creek is located within the Empire Mining District in the Clear Creek Watershed, Colorado, USA (Fig. 1). The stream originates near the Continental Divide in Clear Creek County and flows for approximately 1.4 km into North Empire Creek. The region is underlain by Precambrian rocks that include the Idaho Springs Formation and Boulder Creek and Silver Plume granites (Lovering and Goddard 1950). Ore was discovered in the Empire Mining District in 1862 and mining continued until the mid-1940s. The ore deposits are primarily of the pyritic gold-type and include chalcopyrite, pyrite, and quartz, with smaller quantities of galena and sphalerite. Gold and copper were the chief metal products. Minnesota Mine, to the east of Lion Creek, was once the largest producer of gold in Clear Creek County and includes 2100 m of underground workings on numerous levels. The upper levels of the mine access the Atlantic and Comet veins and are drained by a main portal that is adjacent to a shaft that accessed the lower levels (Lovering and Goddard 1950). At the time of this study, the main portal was collapsed and water leaving the collapsed adit flowed along a short channel before entering a grated concrete chamber situated over the shaft collar. Water levels in the shaft (elevation 2963 m) were 0–2 m below the top of the shaft during the course of this study, indicating that the lower mine levels were completely flooded.

Lion Creek now forms part of the Clear Creek/Central City Superfund Site (EPA ID: COD980717557) due to extensive historical mining operations. Water quality in the Clear Creek watershed is a significant issue as it is a major drinking water source, fishery, and recreation area. The study reach originates just upstream from Minnesota Mine (MN-0000, Fig. 1) and terminates downstream (MN-1469, Fig. 1) from the North Empire Creek confluence, an approximate length of 1469 m. The most obvious effects of mine contamination in the study reach are iron hydroxide precipitates that are visible in the streambed from ∼120 m downstream from site MN-0000. The study reach includes several potential source areas of metals and acidity to Lion Creek. First, the left bank from ∼230 to 300 m is eroding in places and contains numerous small seeps throughout. Upstream from this section (∼60 to 120 m), remedial actions to minimise erosion include hillslope stabilisation with geotextile mesh and rock armouring of a high gradient left bank inflow channel (Holm 2012; left or right as viewed by an observer looking downstream). Second, a seepage face is located from ∼300 to 350 m and has a possible hydraulic connection to the Minnesota Mine shaft. Third, there are large deposits of mine tailings located on the left bank from ∼370 to 400 m. Fourth, several small inflows are located between ∼500 and 600 m. One of these inflows enters along the left bank of Lion Creek (MN-0550, Fig. 1) and site topography suggests that it may receive water from the flooded lower levels of the mine. Fifth, mine waste dumps are located between ∼600 to 900 m associated with Pirate, Raleigh, and Lafayette mines (Fig. 1). Sixth, the confluence with North Empire Creek at 1403 m is a potential source as this creek drains Conqueror Mine which was also a significant producer of ore (Lovering and Goddard 1950).

### Tracer injection and synoptic sampling

Constituent loads were quantified under low flow conditions using the tracer dilution method (to estimate streamflow) and synoptic sampling (to quantify stream and inflow chemistry) (Kimball et al. 2002; Runkel et al. 2013). Two salts were used to make up the tracer injection solution. Sodium bromide (NaBr) was included to estimate flow in the upper portion of the stream where pH is circum-neutral (Br should be conservative at circum-neutral pH) (Dzombak and Morel 1990). Lithium chloride (LiCl) was included to estimate flow in the lower portion of the stream where pH is <3.5 (Li should be conservative at low pH) (Dzombak and Morel 1990). The salts (10.5 kg NaBr and 25 kg LiCl) were dissolved in 100 L of stream water. The resultant injection solution had Br and Li concentrations of 81.3 and 40.8 g/L, respectively. The constant-rate tracer injection was initiated at 18:21 h on Monday 25 August 2014 at a rate of 68 mL/min. The injection was terminated at 13:35 hours on Tuesday 26 August 2014 following completion of the synoptic sampling.

Synoptic samples were collected at 25 stream sites, 15 inflow sites, 5 piezometer (well) sites, and 2 mine water sites (Fig. 1; Table 1) on the morning of 26 August when instream tracer concentrations had reached a steady-state plateau. Inflow sites included visible tributary-type inflows and more ill-defined flows such as riparian seeps and springs. In addition to the synoptic samples, water samples were collected from the Minnesota mine adit (MN-ADIT), an open pool in the Minnesota Mine shaft (MN-POOL), and from subsurface pore waters at the seep face at 324 m (MN-0324A-E equate to 10, 20, 30, 40, 50 cm depth). The latter were sampled by installing hollow 3/8″ stainless steel piezometer drivepoints (with a 1-cm slot opening just above the drivepoint) in the seep face to the desired depths. A peristaltic pump attached to the drivepoint was used to retrieve pore water samples.

**Table 1:** pH, specific conductivity, alkalinity, tracer, and major ion concentrations, Lion Creek, Colorado, August 2014

Site ID	Source	Dist. (m)		μS/cm	mg/L					
			pH	Ksc	Alk.	Cl	Br	F	NO_3_	SO_4_
MN-ADIT	Mine water	0	2.74	2660	NA	0.83	<0.03	9.58	<0.02	1331.26
MN-POOL	Mine water	0	2.88	2420	NA	0.73	<0.03	9.45	<0.02	1263.99
MN-0000	STR	0	6.85	54.1	11.59	0.21	<0.03	0.30	<0.02	9.76
MN-0057	STR	57	6.76	335	11.21	76.89	29.29	0.21	<0.02	10.22
MN-0095	STR	95	6.69	323	11.16	73.19	28.04	0.27	<0.02	10.14
MN-0139	STR	139	6.70	318	9.23	70.29	27.03	0.25	<0.02	13.27
MN-0174	STR	174	6.69	313	7.98	68.01	26.41	0.25	<0.02	15.56
MN-0181	LBI	181	3.06	1250	NA	0.93	<0.03	3.93	0.44	545.64
MN-0191	STR	191	5.10	339	NA	64.05	25.33	0.39	<0.02	37.78
MN-0232	STR	232	4.62	384	NA	59.09	23.39	0.78	<0.02	67.94
MN-0258	LBI	258	2.68	2460	NA	0.90	<0.03	5.53	<0.02	1159.18
MN-0270	LBI	270	2.63	2530	NA	1.23	<0.03	4.59	<0.02	1144.77
MN-0276	STR	276	3.92	482	NA	54.90	21.80	0.92	<0.02	111.40
MN-0286	LBI	286	2.76	2710	NA	1.04	<0.03	4.26	<0.02	1408.84
MN-0300	STR	300	3.68	569	NA	52.20	20.74	0.99	<0.02	145.85
MN-0300B	STR	300	3.69	567	NA	52.27	20.71	0.95	<0.02	143.20
MN-0317	LBI	317	2.60	2830	NA	1.13	<0.03	7.48	<0.02	1317.39
MN-0318	RBI	318	2.84	2260	NA	0.81	0.61	7.36	<0.02	1114.60
MN-0324	LBI	324	2.55	2820	NA	0.83	<0.03	8.90	<0.02	1254.77
MN-0324A	LBP	324	2.52	2970	NA	1.23	<0.03	8.83	<0.02	1351.61
MN-0324B	LBP	324	2.57	2770	NA	0.97	<0.03	8.31	<0.02	1338.95
MN-0324C	LBP	324	2.62	2870	NA	1.04	<0.03	7.83	<0.02	1327.04
MN-0324D	LBP	324	2.67	2880	NA	1.06	<0.03	6.94	<0.02	1497.38
MN-0324E	LBP	324	2.69	2840	NA	1.04	<0.03	7.49	<0.02	1339.74
MN-0344	STR	344	3.09	971	NA	43.84	16.99	1.27	<0.02	294.58
MN-0345	RBI	345	2.65	2110	NA	0.81	<0.03	6.55	<0.02	808.10
MN-0370	STR	370	3.09	999	NA	42.64	17.07	1.19	<0.02	290.24
MN-0370B	STR	370	3.09	996	NA	43.07	17.14	1.22	<0.02	306.30
MN-0387	RBI	387	6.67	38.3	10.76	0.38	<0.03	0.20	<0.02	4.64
MN-0398	STR	398	3.11	965	NA	39.46	15.60	1.28	<0.02	293.40
MN-0403	RBI	403	2.93	1393	NA	0.72	0.62	5.09	<0.02	511.98
MN-0425	STR	425	3.17	983	NA	36.47	14.48	1.28	<0.02	320.05
MN-0466	STR	466	3.14	1004	NA	35.50	14.04	1.20	<0.02	323.80
MN-0470	RBI	470	6.88	50.2	15.87	0.38	<0.03	0.23	<0.02	5.51
MN-0491	STR	491	3.15	969	NA	0.62	<0.03	0.62	<0.02	311.65
MN-0507	RBI	507	3.67	360	NA	33.80	13.42	1.30	<0.02	136.03
MN-0545	STR	545	3.14	1033	NA	31.97	12.67	1.21	<0.02	356.96
MN-0550	LBI	550	2.66	2870	NA	1.90	<0.03	6.32	<0.02	1535.56
MN-0579	STR	579	3.06	1214	NA	29.32	11.22	0.90	<0.02	439.33
MN-0656	STR	656	3.15	1224	NA	27.93	10.73	0.91	<0.02	457.01
MN-0750	STR	750	3.15	1218	NA	27.74	10.61	0.91	<0.02	450.95
MN-0868	STR	868	3.14	1212	NA	27.03	10.44	0.88	<0.02	448.10
MN-0969	STR	969	3.14	1198	NA	25.34	9.71	0.94	<0.02	445.66
MN-0995	RBI	995	6.52	60.1	15.02	0.75	<0.03	0.27	<0.02	8.34
MN-1100	STR	1100	3.08	1164	NA	23.34	9.21	0.91	<0.02	417.11
MN-1245	STR	1245	3.16	1134	NA	21.83	8.80	1.01	<0.02	416.09
MN-1381	STR	1381	3.08	1135	NA	21.09	8.32	0.93	<0.02	413.27
MN-1403	LBI	1403	4.26	928	NA	4.75	<0.03	0.87	<0.02	403.34
MN-1469	STR	1469	3.27	1044	NA	14.13	4.90	1.08	0.02	408.47

Distance along study reach, in meters (m), alkalinity as CaCO_3_; MN-0300B and MN-0370B are part of field replicates

*NA* not analysed, *STR* stream sample, *LBI* left bank inflow, *RBI* right bank inflow, *LBP* left bank piezometer

Water temperature was measured in situ using an alcohol thermometer. Samples were transported to a central processing area where 125 mL aliquots were prepared for cation and anion analyses. On-site processing included filtration (0.45 μm), measurement of pH and specific conductance, and preservation of samples for iron speciation. Anion concentrations were determined from filtered, unacidified samples by ion chromatography. Anion concentrations are reported for Cl, Br, F, NO_3_, and SO_4_ (Table 1). Aliquots for cation analysis were acidified to pH <2 with ultrapure HNO_3_. Total recoverable and dissolved (some colloidal material may have passed through the 0.45-μm filter) cation concentrations were determined from unfiltered and filtered samples, respectively, by inductively coupled plasma–mass spectroscopy (ICP-MS). Trace element concentrations are reported for Al, Ag, As, Ca, Cu, Ba, Cd, Co, Cr, Fe, K, Li, Mg, Mn, Mo, Na, Ni, Pb, Si, Sr, U, V, and Zn (Table 2). Alkalinity was determined from filtered, unacidified samples. Aquatic life standards (Colorado Department of Public Health and Environment 2005) for all metals were calculated based on the water hardness of each sample. Patterns in major ion chemistry were illustrated using a piper diagram in the software GW Chart (Winston 2000).

**Table 2:** Dissolved concentrations of metals, Lion Creek, Colorado, August 2014

Site ID	Source	Dist. (m)	ng/L		μg/L									mg/L												
			Ag	As	Ba	Cd	Co	Cr	Mo	Ni	Pb	U	V	Al	Ca	Cu	Fe	FeII	K	Li	Mg	Mn	Na	Si	Sr	Zn
MN-ADIT	Mine water	0	21.02	140.23	0.83	8.28	308.75	12.96	0.08	232.30	0.08	104.46	13.83	27.39	120.20	5.48	331.71	305.00	0.76	0.05	48.88	23.52	6.86	19.81	253.75	1.78
MN-POOL	Mine water	0	16.04	274.86	7.17	7.44	283.35	10.53	0.08	213.30	0.59	90.13	10.03	23.74	122.08	4.54	314.01	278.00	1.31	0.04	48.69	21.88	6.45	18.07	284.67	1.45
MN-0000	STR	0	<10	26.52	9.86	0.10	<0.1	0.04	0.19	0.41	0.01	0.14	0.08	0.01	4.28	0.00	0.01	0.02	0.45	<0.008	0.90	0.00	2.83	6.57	41.54	0.01
MN-0057	STR	57	<10	152.10	14.82	0.12	<0.1	0.04	0.40	0.42	0.03	0.11	0.07	<0.007	5.03	0.00	0.01	0.01	0.55	14.73	1.06	0.00	10.29	6.87	46.62	0.01
MN-0095	STR	95	<10	42.01	15.76	0.10	<0.1	0.05	0.39	0.42	0.03	0.12	0.07	<0.007	5.49	0.00	0.01	0.01	0.58	13.81	1.16	0.00	9.71	6.82	51.54	0.01
MN-0139	STR	139	<10	65.67	15.67	0.05	0.56	0.07	0.36	0.95	0.06	0.26	0.06	0.08	5.87	0.01	0.12	0.04	0.60	13.33	1.29	0.05	9.71	7.01	54.01	0.02
MN-0174	STR	174	<10	99.51	16.06	0.18	1.05	0.03	0.33	1.30	0.00	0.24	0.04	0.03	6.35	0.01	0.02	0.04	0.60	12.43	1.43	0.07	9.15	6.91	58.39	0.02
MN-0181	LBI	181	22.58	23.20	1.85	5.29	170.21	2.12	0.01	107.10	0.14	96.68	0.03	44.76	59.33	1.20	3.54	0.27	1.66	0.05	26.00	9.58	7.45	24.20	336.00	1.02
MN-0191	STR	191	36.15	24.69	15.90	0.35	7.22	0.04	0.23	6.06	0.11	3.14	0.01	0.78	8.93	0.06	0.05	0.04	0.68	11.99	2.59	0.44	9.32	7.67	69.23	0.06
MN-0232	STR	232	<10	94.49	15.80	0.50	14.28	0.11	0.06	10.52	0.15	6.68	<0.006	2.58	13.42	0.09	0.13	0.11	0.76	11.26	4.14	1.06	10.01	9.15	87.88	0.10
MN-0258	LBI	258	18.90	123.46	4.14	10.99	435.82	8.08	0.12	244.85	0.37	106.31	0.37	64.84	121.21	1.63	73.21	14.20	0.71	0.08	49.96	30.94	10.46	51.99	481.72	1.58
MN-0270	LBI	270	20.97	385.81	3.79	9.46	413.30	10.53	0.21	234.74	0.21	154.24	1.46	72.52	96.60	2.10	68.24	1.52	0.04	0.08	48.04	25.38	9.77	52.87	327.08	1.62
MN-0276	STR	276	<10	52.26	15.05	1.05	33.77	0.39	0.02	21.27	0.20	11.18	<0.006	5.08	18.76	0.15	1.10	0.45	0.80	10.43	6.22	2.64	9.67	10.76	105.53	0.17
MN-0286	LBI	286	32.95	260.63	3.97	16.08	993.95	5.00	0.13	317.30	0.52	203.81	3.15	103.02	150.98	2.78	36.12	20.10	1.85	0.10	58.31	53.18	11.69	57.54	613.43	2.03
MN-0300	STR	300	<10	38.06	15.10	1.55	71.22	0.67	0.04	34.59	0.22	16.12	0.01	7.68	21.93	0.23	2.27	1.10	0.84	10.24	7.71	4.39	9.20	11.93	129.09	0.23
MN-0300B	STR	300	<10	25.65	15.69	1.53	71.94	0.65	0.05	34.03	0.24	15.85	0.01	7.52	21.53	0.24	2.27	1.00	0.81	10.14	7.63	4.34	9.01	11.78	130.77	0.23
MN-0317	LBI	317	13.44	40.50	0.45	11.27	556.37	7.62	0.03	278.82	0.23	109.54	1.18	55.67	129.72	2.28	134.36	84.90	0.35	0.06	61.31	36.94	10.02	44.60	372.10	1.68
MN-0318	RBI	318	34.77	109.26	4.61	19.82	1228.52	5.78	0.07	309.16	0.59	142.80	0.16	73.31	127.80	2.66	19.87	1.26	1.54	0.29	49.31	64.64	9.93	44.21	531.44	2.14
MN-0324	LBI	324	18.87	32.95	0.87	7.14	353.35	7.25	0.04	239.42	0.25	125.73	0.16	49.88	128.48	2.37	104.79	0.73	0.13	0.06	57.88	25.41	9.12	39.39	353.27	1.37
MN-0324A	LBP	324	14.98	59.72	0.86	7.22	363.66	10.14	0.05	254.26	0.34	110.24	0.23	58.65	145.64	2.32	130.95	11.60	0.22	0.06	64.76	29.58	11.03	44.95	359.01	1.60
MN-0324B	LBP	324	14.53	60.37	1.49	7.84	398.43	10.08	0.05	258.13	0.33	108.90	0.47	56.95	137.61	2.29	147.11	53.20	0.30	0.07	61.22	30.34	10.69	44.96	371.41	1.59
MN-0324C	LBP	324	19.04	59.54	1.66	9.21	484.07	14.15	0.07	267.23	0.47	106.80	0.89	58.73	132.97	2.22	186.39	161.00	0.51	0.07	60.93	33.77	10.85	44.01	406.57	1.62
MN-0324D	LBP	324	16.33	139.64	2.40	15.89	557.46	44.55	0.09	282.35	1.16	113.50	2.11	68.21	146.55	2.34	227.82	226.00	0.70	0.07	68.95	41.10	12.44	51.62	407.66	1.88
MN-0324E	LBP	324	15.89	38.06	2.26	10.17	548.29	10.32	0.04	277.76	0.26	103.33	1.19	59.52	134.57	2.22	202.48	204.00	0.59	0.07	59.79	37.64	10.87	46.29	400.56	1.70
MN-0344	STR	344	<10	58.29	12.72	2.72	134.09	1.67	0.10	68.19	0.25	30.07	0.15	14.42	37.50	0.52	15.53	2.69	0.78	8.41	14.65	8.58	9.05	16.17	164.09	0.44
MN-0345	RBI	345	13.15	37.27	6.68	7.18	339.55	7.97	0.04	180.32	1.34	82.19	0.94	35.99	83.08	1.51	50.19	11.70	1.18	0.05	39.88	22.11	7.66	31.31	265.86	1.06
MN-0370	STR	370	<10	96.02	12.71	3.18	142.90	1.85	0.11	73.03	0.43	31.41	0.18	14.69	40.06	0.54	16.48	3.95	0.85	8.40	15.64	9.22	9.47	15.97	168.02	0.48
MN-0370B	STR	370	<10	22.53	12.69	3.06	140.07	1.63	0.10	69.86	0.31	30.37	0.15	15.05	38.80	0.53	16.32	3.88	0.79	8.20	15.26	8.89	9.14	16.70	169.17	0.46
MN-0387	RBI	387	<10	24.73	10.51	<0.01	0.15	0.06	2.58	0.08	0.05	0.10	0.11	0.04	2.68	0.00	0.07	0.05	0.54	<0.008	0.72	<0.002	2.64	6.56	22.19	<0.001
MN-0398	STR	398	<10	43.08	13.50	2.90	137.88	1.53	0.16	69.90	0.31	28.55	0.23	14.32	37.93	0.49	16.62	5.54	0.79	7.51	14.74	8.88	8.56	16.03	172.33	0.44
MN-0403	RBI	403	<10	23.41	11.31	3.18	249.17	0.93	0.03	123.81	0.36	23.42	0.14	23.75	60.13	0.26	33.01	16.10	1.30	0.43	23.84	18.47	6.05	21.72	215.64	0.76
MN-0425	STR	425	<10	87.31	13.11	3.09	140.93	1.59	0.19	71.37	0.28	28.94	0.29	15.50	41.08	0.48	18.18	7.72	0.86	6.91	16.51	9.94	9.03	16.95	182.49	0.48
MN-0466	STR	466	<10	121.83	12.36	3.06	146.80	1.68	0.17	76.00	0.32	30.84	0.26	16.25	41.26	0.50	17.87	9.24	0.81	6.76	16.11	9.80	8.49	17.23	166.62	0.49
MN-0470	RBI	470	<10	59.95	22.35	0.01	<0.1	0.06	2.57	0.26	0.05	0.14	0.13	0.02	4.03	0.00	0.05	0.06	0.57	<0.008	1.15	0.01	3.01	7.09	31.65	0.00
MN-0491	STR	491	<10	38.53	13.14	3.03	145.23	1.61	0.24	74.79	0.34	30.43	0.24	15.71	40.12	0.49	16.62	9.25	0.81	6.60	15.62	9.43	8.26	16.77	168.05	0.47
MN-0507	RBI	507	<10	47.25	33.32	1.60	40.80	0.39	0.03	31.38	0.61	8.62	0.07	5.14	19.69	0.11	3.66	3.47	0.99	0.01	7.38	3.98	4.66	10.63	75.29	0.21
MN-0545	STR	545	10.46	43.90	12.73	3.52	154.59	1.84	0.22	83.98	0.33	35.59	0.19	18.72	47.94	0.54	18.06	6.02	0.88	6.17	18.43	11.54	8.98	18.34	178.96	0.59
MN-0550	LBI	550	25.62	34.35	2.69	19.24	630.52	18.17	0.07	450.34	0.32	278.15	0.12	107.60	121.41	4.40	62.66	1.21	0.27	0.11	91.62	36.22	10.05	49.35	332.76	2.85
MN-0579	STR	579	13.46	53.69	12.60	5.06	200.02	3.02	0.23	118.97	0.35	56.13	0.20	25.83	52.91	0.83	19.75	5.39	0.83	5.57	23.91	13.22	8.41	20.69	203.02	0.92
MN-0656	STR	656	14.17	100.28	11.61	5.64	207.02	2.99	0.20	125.10	0.32	58.27	0.17	27.09	54.32	0.87	18.49	4.58	0.89	5.34	25.02	13.38	8.47	21.38	195.23	0.94
MN-0750	STR	750	15.32	76.52	12.17	5.25	198.50	3.12	0.19	121.70	0.30	55.51	0.16	26.81	54.08	0.84	17.83	4.15	0.87	5.14	24.55	13.30	8.33	20.92	201.95	0.93
MN-0868	STR	868	12.49	81.78	11.74	5.12	200.26	2.82	0.17	122.72	0.31	56.51	0.14	26.88	53.69	0.85	17.49	3.66	0.89	5.07	24.58	13.25	8.31	21.12	198.75	0.94
MN-0969	STR	969	16.41	64.91	11.67	4.99	194.21	2.90	0.15	119.63	0.29	54.08	0.13	26.35	53.75	0.82	16.23	2.47	0.93	4.75	24.27	13.04	8.30	21.12	197.08	0.91
MN-0995	RBI	995	<10	45.45	27.56	0.05	<0.1	0.05	4.57	0.29	0.07	0.05	0.06	0.03	4.68	0.00	0.02	0.01	0.44	0.01	1.28	<0.002	3.19	8.08	45.69	0.00
MN-1100	STR	1100	16.84	38.86	12.94	4.85	186.24	2.76	0.20	114.30	0.32	53.13	0.11	24.93	50.99	0.78	15.01	2.07	0.90	4.47	23.27	12.35	7.95	20.26	202.80	0.87
MN-1245	STR	1245	18.18	71.02	13.34	5.06	186.11	2.55	0.16	112.44	0.37	51.42	0.09	24.50	50.42	0.78	14.06	1.51	0.95	4.28	22.70	12.04	7.82	20.32	186.22	0.85
MN-1381	STR	1381	20.66	61.65	14.07	5.01	185.15	2.74	0.16	112.40	0.46	51.06	0.09	24.47	50.50	0.77	12.14	1.23	0.98	4.14	22.64	12.16	7.78	20.27	202.71	0.72
MN-1403	LBI	1403	<10	459.93	34.34	1.36	88.80	0.16	0.01	102.59	1.28	2.53	0.01	1.52	73.61	0.16	37.14	40.00	5.19	0.01	30.62	12.63	8.88	12.27	330.65	0.32
MN-1469	STR	1469	<10	272.37	21.41	3.48	144.26	1.66	0.06	108.08	1.14	30.92	0.03	15.30	59.00	0.52	21.21	15.80	2.63	2.39	25.79	12.24	8.20	17.13	246.42	0.56

Distance along study reach, in meters (m); MN-0300B and MN-0370B are part of field replicates

*STR* stream sample, *LBI* left bank inflow, *RBI* right bank inflow, *LBP* left bank piezometer

### Estimating streamflow

**Fig. 2:**
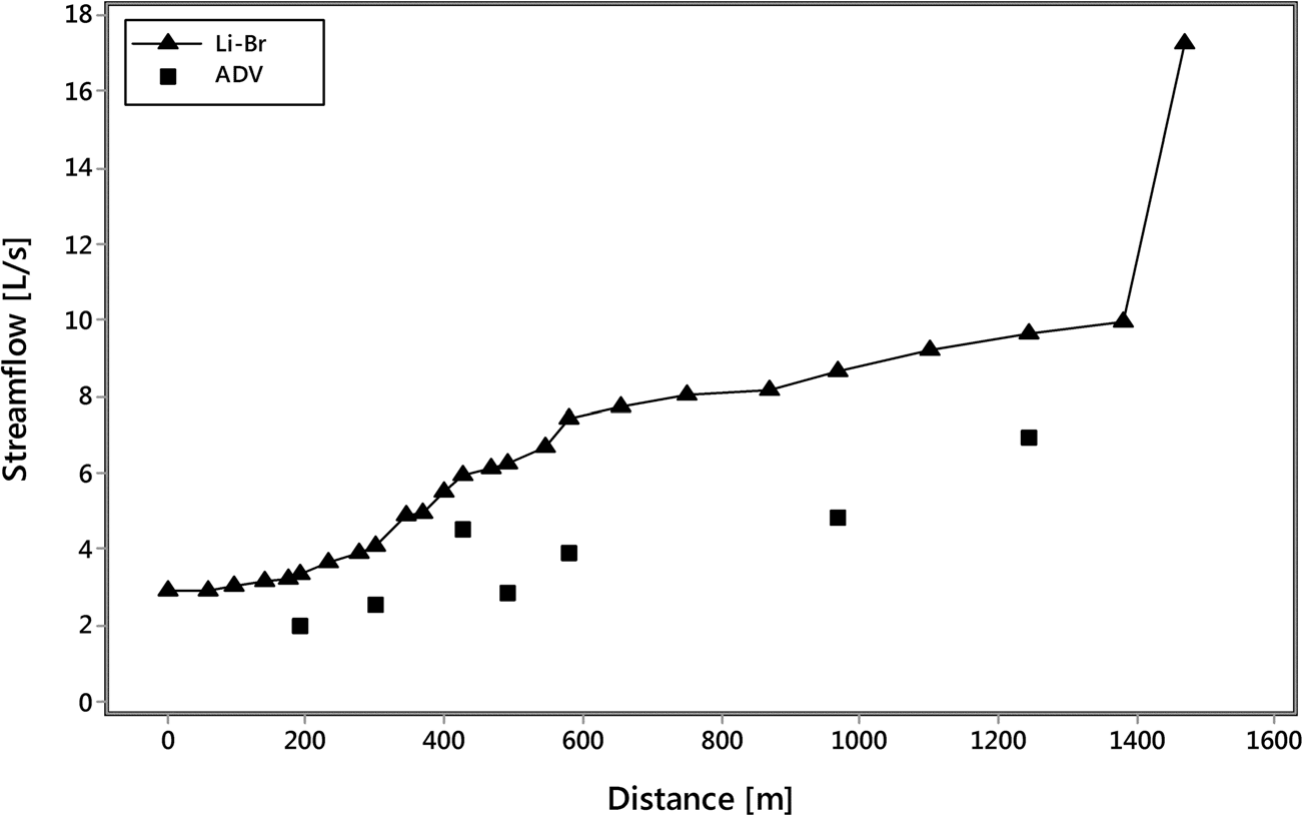
Streamflow estimates derived from the tracer dilution method showing the final Li-Br hybrid streamflow estimate. Streamflow estimates from acoustic Doppler velocity (ADV) measurements are also shown

Quantification of discharge by tracer dilution method is ideal in high gradient, mountainous streams like Lion Creek where irregular channel bottoms and hyporheic flow compromise traditional methods such as velocity-area flow estimation. Dilution of an injected tracer overcomes these issues as the tracer mixes completely with the stream water and follows subsurface flow paths. Once the tracer reaches a plateau concentration, synoptic samples can be collected in order to characterise changes of streamflow of only a few percent (Kimball et al. 2002). Decreases in plateau concentration with stream length reflect dilution of the tracer as surface drainage and/or groundwater inputs result in increased streamflow. Calculation of streamflow (*Q*) at each synoptic site relates the injected tracer to the observed dilution at the site (Kilpatrick and Cobb 1985):

1 $$ Q={Q}_{\mathrm{INJ}}{C}_{\mathrm{INJ}}/\left({C}_{\mathrm{P}}\hbox{--} {C}_{\mathrm{B}}\right) $$

where *Q*_INJ_ is the injection rate, *C*_INJ_ is the tracer injectate concentration, *C*_P_ is the tracer plateau concentration at the synoptic site, and *C*_B_ is the tracer background concentration in the stream water. Three flow profiles were initially generated using the equation above for the observed Br, Cl, and Li concentrations (Fig. S1). These three profiles are in general agreement, with the Li flow profile having the highest flow values. A final flow profile (Fig. 2) was generated using a hybrid of the Br and Li flow profiles:

1. Sites MN-0000 to MN-0344—flow at these sites was calculated using the observed Br concentrations and Eq. 1 above. pH is circum-neutral in this subreach, so Br is assumed to behave conservatively.
2. Sites MN-0370 to MN-1469. pH is <3.5 in this subreach. Although Br appears to be conservative (the three profiles noted above are in general agreement), there may be small losses of Br at low pH. Li, in contrast, should be conservative at these pH values. Streamflow at the downstream site in a site pair (*Q*_d_) was therefore calculated based on the observed Li dilution:

2 $$ {Q}_{\mathrm{d}}=\left({C}_{\mathrm{u}}/{C}_{\mathrm{d}}\right)\times {Q}_{\mathrm{u}} $$

where *C* is the plateau Li concentration and u and d denote upstream and downstream sites, respectively.

Streamflow estimates from the tracer dilution method are compared to streamflow estimates from acoustic Doppler velocity measurements (Turnipseed and Sauer 2010) in Fig. 2.

### Loading analysis

The study reach was divided into 24 stream segments demarcated by the 25 stream sampling sites (Fig. 1, Table 3). Constituent loads were calculated as the product of tracer-derived discharge and constituent concentration. Dissolved concentrations are used in the loading analysis due to the close correspondence between total recoverable and dissolved constituents (Runkel et al. 2013).

**Table 3:** Summary of gain and loss of constituent loads in Lion Creek, Colorado, August 2014

Stream segment	Al	Co	Cd	Cu	Fe	Mn	Ni	SO_4_	Zn
1: 0–57 m	−0.03	0.00	0.000	0.00	−0.01	0.00	0.00	1.35	0.00
2: 57–95 m	0.00	0.00	0.000	0.00	0.01	0.00	0.00	1.09	0.01
3: 95–139 m	0.23	0.00	0.000	0.03	0.35	0.15	0.00	11.02	0.02
4: 139–174 m	−0.16	0.00	0.000	0.00	−0.34	0.06	0.00	8.36	0.01
5: 174–191 m	2.52	0.02	0.001	0.19	0.10	1.26	0.02	76.96	0.15
6: 191–232 m	6.80	0.03	0.001	0.11	0.32	2.39	0.02	120.44	0.16
7: 232–276 m	10.45	0.08	0.002	0.25	3.85	6.43	0.04	187.89	0.31
8: 276–300 m	11.40	0.16	0.002	0.39	5.02	7.64	0.06	158.86	0.28
9: 300–344 m	39.39	0.36	0.007	1.58	66.81	24.09	0.19	849.58	1.19
10: 344–370 m	3.17	0.05	0.002	0.11	5.29	2.92	0.02	37.17	0.19
11: 370–398 m	4.78	0.05	0.000	0.04	9.78	3.80	0.03	129.05	0.06
12: 398–425 m	13.85	0.08	0.003	0.18	17.24	10.58	0.04	299.52	0.50
13: 425–466 m	6.67	0.05	0.000	0.18	0.58	0.47	0.04	65.74	0.10
14: 466–491 m	−1.04	0.01	0.000	0.02	−5.26	−0.88	0.00	−29.03	−0.05
15: 491–545 m	27.00	0.13	0.005	0.53	16.95	18.25	0.09	439.82	1.03
16: 545–579 m	66.16	0.45	0.014	2.55	9.57	20.71	0.32	866.41	1.72
17: 579–656 m	17.94	0.12	0.006	0.58	−1.50	5.48	0.09	277.09	0.50
18: 656–750 m	6.03	−0.01	−0.001	0.03	1.60	3.42	0.01	90.07	0.15
19: 750–868 m	3.42	0.04	0.000	0.12	−2.85	1.01	0.02	25.42	0.07
20: 868–969 m	10.01	0.06	0.002	0.20	1.35	5.38	0.04	222.06	0.35
21: 969–1100 m	1.11	0.03	0.001	0.11	−3.35	0.70	0.02	−22.52	0.00
22: 1100–1245 m	6.44	0.08	0.004	0.37	−3.15	2.21	0.03	166.67	0.23
23: 1245–1381 m	7.32	0.05	0.001	0.06	−1.53	4.92	0.03	101.71	0.19
24: 1381–1469 m	20.48	0.65	0.010	1.33	245.25	90.29	0.75	2935.98	2.48

All loading values in milligram/second, stream segment in meters

Cumulative instream load is equal to the sum of all increases in constituent load (Kimball et al. 2002). For a given stream segment, the cumulative instream load is increased if the constituent load increased, and held constant if the constituent load decreased. The cumulative instream load provides an estimate of the total constituent load added to the stream over the entire study reach whereas the constituent loading represents the net amount of loading after chemical reaction such as adsorption and precipitation. A net increase in cumulative instream load suggests addition of constituent mass to the stream. The percent contribution of each source is given by:

4 $$ \%\mathrm{contribution}=100\times \Delta \mathrm{load}/\left(\mathrm{L}2\hbox{--} \mathrm{L}1\right) $$

where Δload is the within-segment increase, and L1 and L2 are the cumulative instream loads at the upstream and downstream ends of the study reach, respectively. Percent contributions from multiple segments were grouped to represent the total contributions from the main source areas within Lion Creek. Comparison of cumulative instream load with total instream load provides a means of estimating net attenuation of constituents over the length of the study reach.

Spatial profiles of constituent load provide a means to calculate ‘effective inflow concentrations’ for stream segments exhibiting an increase in constituent load. Effective inflow concentration represents the average constituent concentration entering a stream segment via surface or subsurface flow under the assumption of conservative transport, i.e. the inflow concentration that accounts for the observed increase in instream constituent load (n.b., the assumption of conservative transport is appropriate herein given the acidic nature of Lion Creek and most inflows). Effective inflow concentrations (*C*_L_) may be developed using simple mass balance calculations on individual stream segments, yielding (Kimball et al. 2002):

$$ {C}_{\mathrm{L}}=\left({Q}_{\mathrm{d}}{C}_{\mathrm{d}}-{Q}_{\mathrm{u}}{C}_{\mathrm{u}}\right)/\left({Q}_{\mathrm{d}}-{Q}_{\mathrm{u}}\right) $$

where *Q* is discharge, *C* is concentration, and u, d, and L represent upstream, downstream, and lateral inflow values, respectively. Effective inflow concentrations calculated in this manner may be used to determine if an observed inflow in a given stream segment is representative of all inflow waters entering the segment. If observed inflow concentrations exceed effective inflow concentrations, there are likely more dilute inflow waters entering the stream segment in addition to the sampled inflow. Conversely, high concentration waters are entering the stream segment when effective inflow concentrations exceed observed inflow concentrations.

### Principal components analysis

Patterns in the chemistry of the stream inflow, mine water, and pore water samples were investigated further using PCA, a multivariate statistical analysis. PCA is an ordination method that aims to reduce a large number of measured variables down to a smaller number of principal components that summarise the information provided by each contributing variable. This method allows the important variables and patterns in multivariate data to be summarised and visualised more easily. Analyses were performed in the program SPSS (https://www.ibm.com/analytics/us/en/technology/spss/). All constituents (dissolved concentrations) except tracer variables (Li, Br, Na, Cl) were included in the analysis. Data were log_10_ (*x* + 1) transformed before analysis to reduce the clustering of common and abundant measurements at the centre of the ordination plot and also the effect of outliers. All constituent concentrations (including pH) were changed to units of millimoles per litre.

Study data are available in the Supplementary Information and the USGS National Water Information System (10.5066/F7P55KJN).

## Results

The following sections focus on stream pH and nine constituents (Al, Cu, Fe, Mn, Zn, Ni, Cd, Co, and SO_4_) that are common to mine drainage and identified in subsequent analyses as a potential cause for concern in Lion Creek.

### Streamflow, pH, and constituent concentrations

Streamflow estimates increase linearly with distance from ∼3 L/s just downstream from the tracer injection site to ∼17 L/s just downstream from the confluence with North Empire Creek (Fig. 2). Aside from the North Empire Creek inflow which contributes 51% to overall streamflow in the study reach, surface inflows are mostly very small tributaries and seeps that are distributed between the left bank (*n* = 8) and right bank (*n* = 7). Only two stream segments contribute more than 5% to overall flow. Segment 300 to 344 m contributes 5.5% to overall streamflow and contains three distinct inflows that includes the seepage face at 324 m. Segment 545 to 579 m contributes 5.1% to streamflow due to two small left bank inflows at 550 m. Only 12 out of the 20 stream segments had identifiable inflows and these segments accounted for 84% of the total streamflow, suggesting some unidentified or unknown dispersed surface and/or subsurface water inputs.

**Fig. 3:**
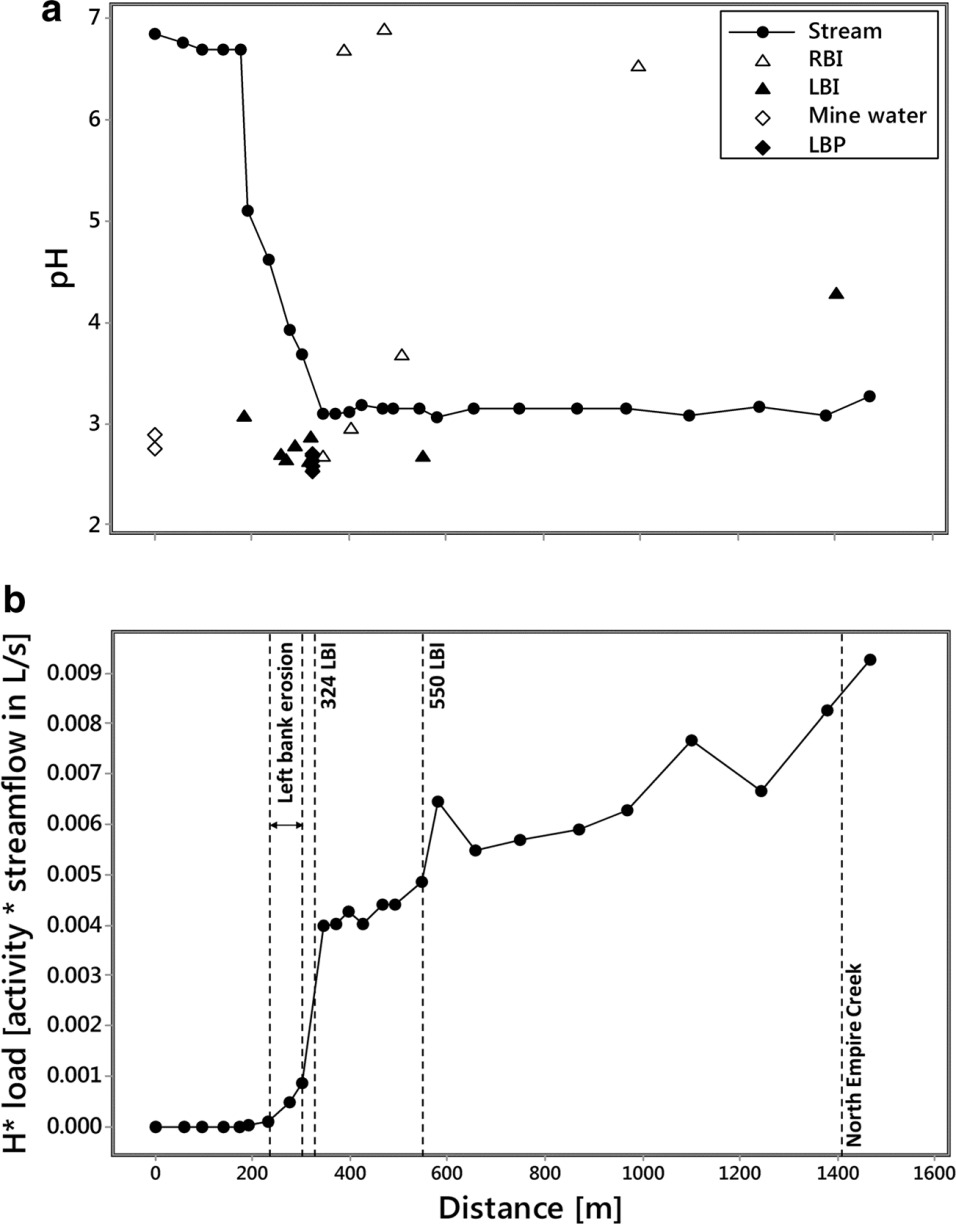
**a** Spatial profile of pH at stream, inflow (*RBI* = right bank inflow; *LBI* = left bank inflow), mine water, and piezometer (*LBP* = left bank piezometer) sites. **b** Spatial profile of H* load based on measurements of instream pH

**Fig. 4:**
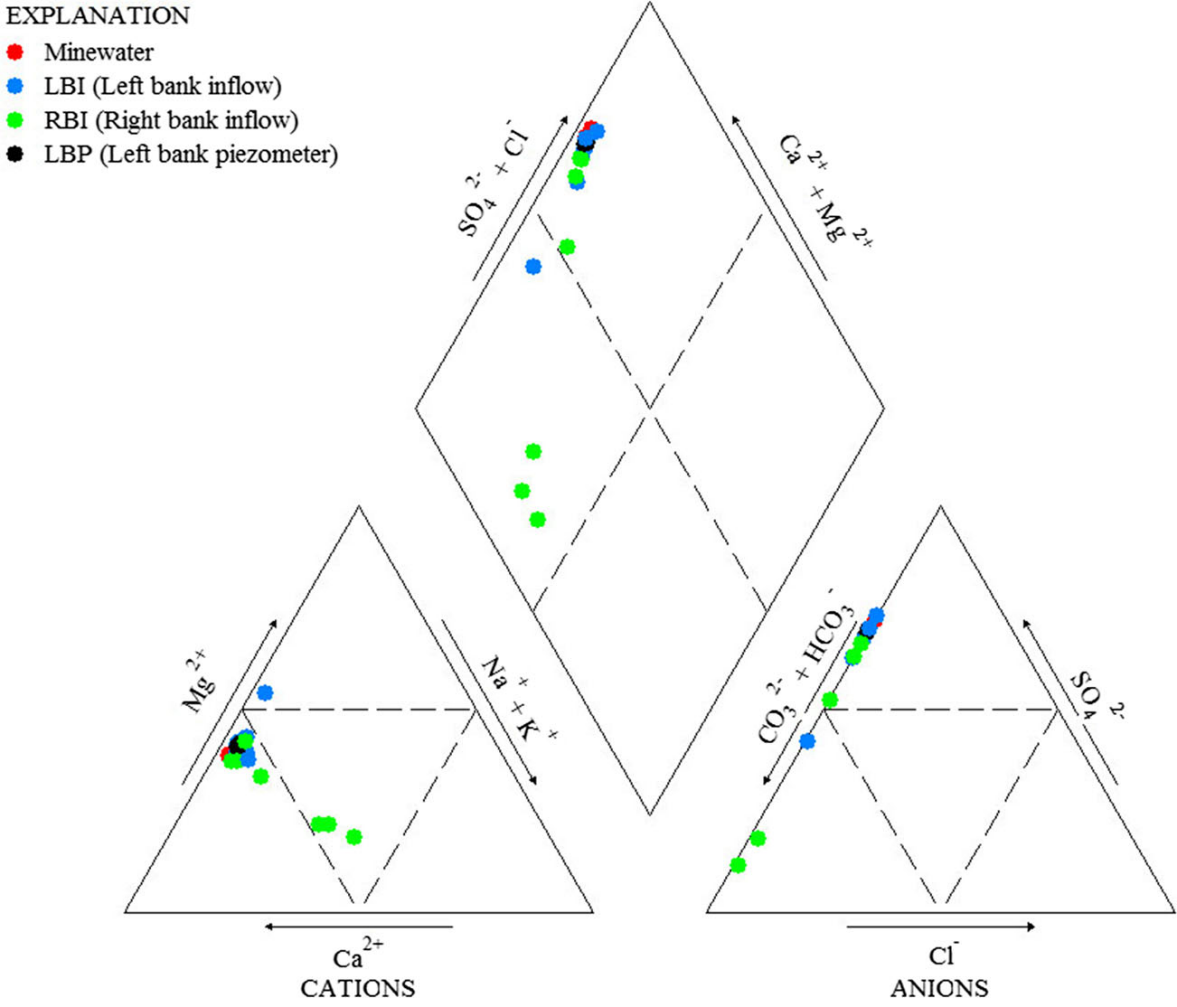
Piper plot illustrating patterns in the major ion chemistry of stream inflow samples

Spatial profiles of pH for stream sites, inflows, piezometers, and mine waters are illustrated in Fig. 3. Stream pH values are generally greater than 6 at the top of the study reach but exhibit a sharp decrease after 174 m that settles out to a pH of ∼3.1 at 370 m. This low stream pH is maintained to the confluence with North Empire Creek. The abrupt decrease in stream pH appears to be related to numerous inflows located between 174 and 370 m that all have pH values in the range 2.5–2.8. These are mostly left bank inflows (MN-0181, MN-0258, MN-0270, MN-0286, MN-0317, MN-0324) but include two right bank inflows (MN-0318, MN-0345). The pH range of these inflows is very similar to piezometer samples (MN-0324A-E) and mine water samples (MN-ADIT, MN-POOL) also entering the stream on the left bank. Beyond 370 m, four further inflows can be considered acidic: two left bank inflows (MN-0550, MN-1403) and two right bank inflows (MN-0403, MN-0507). Probable clean sources of water with circum-neutral pH and located on the right bank are MN-0387, MN-0470, and MN-0995. Examination of patterns in major ion chemistry of the inflow samples illustrates that the left bank samples are predominantly Ca-SO_4_-type and the right bank samples are mostly Ca-HCO_3_-type (Fig. 4).

**Fig. 5:**
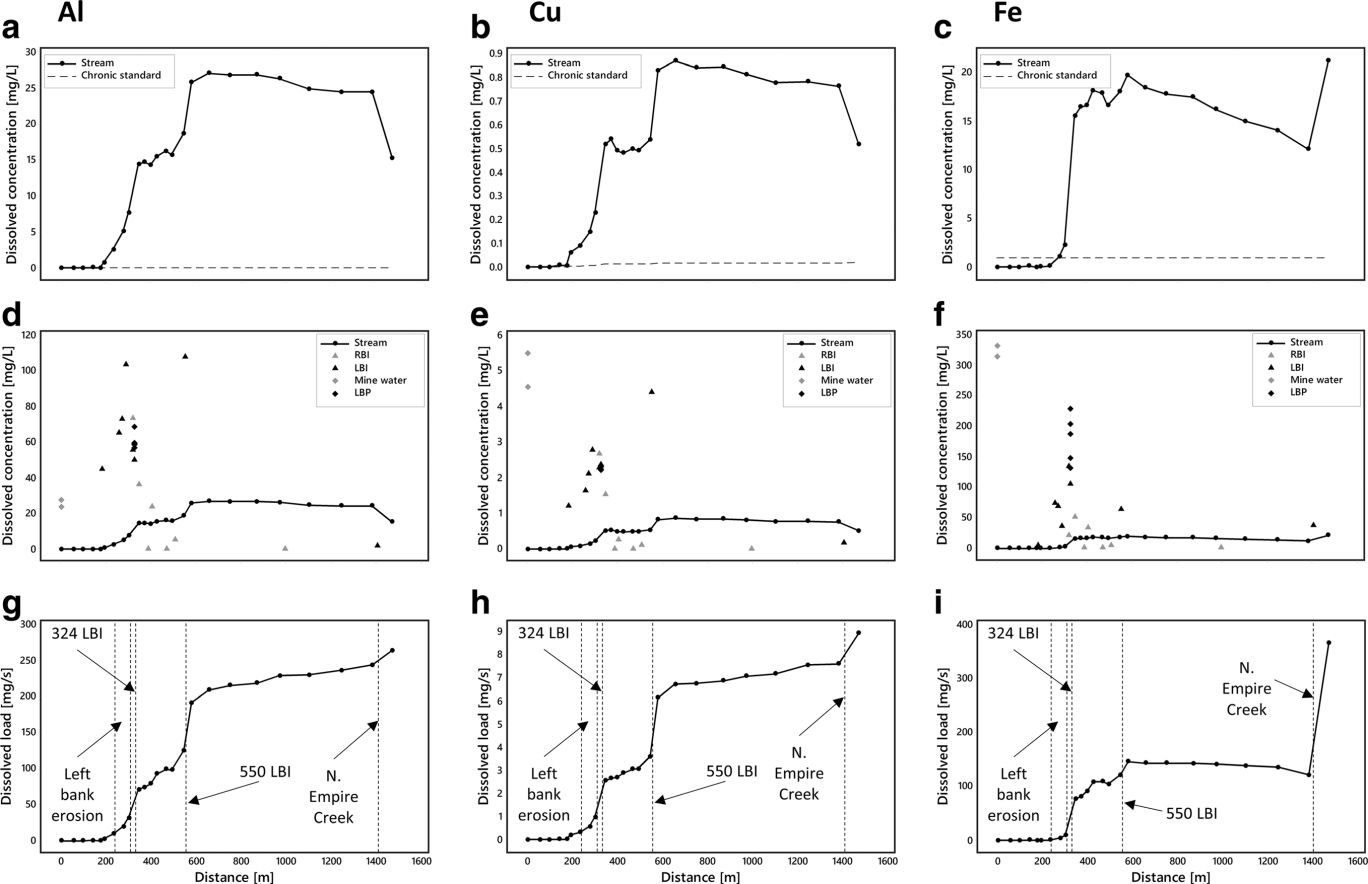

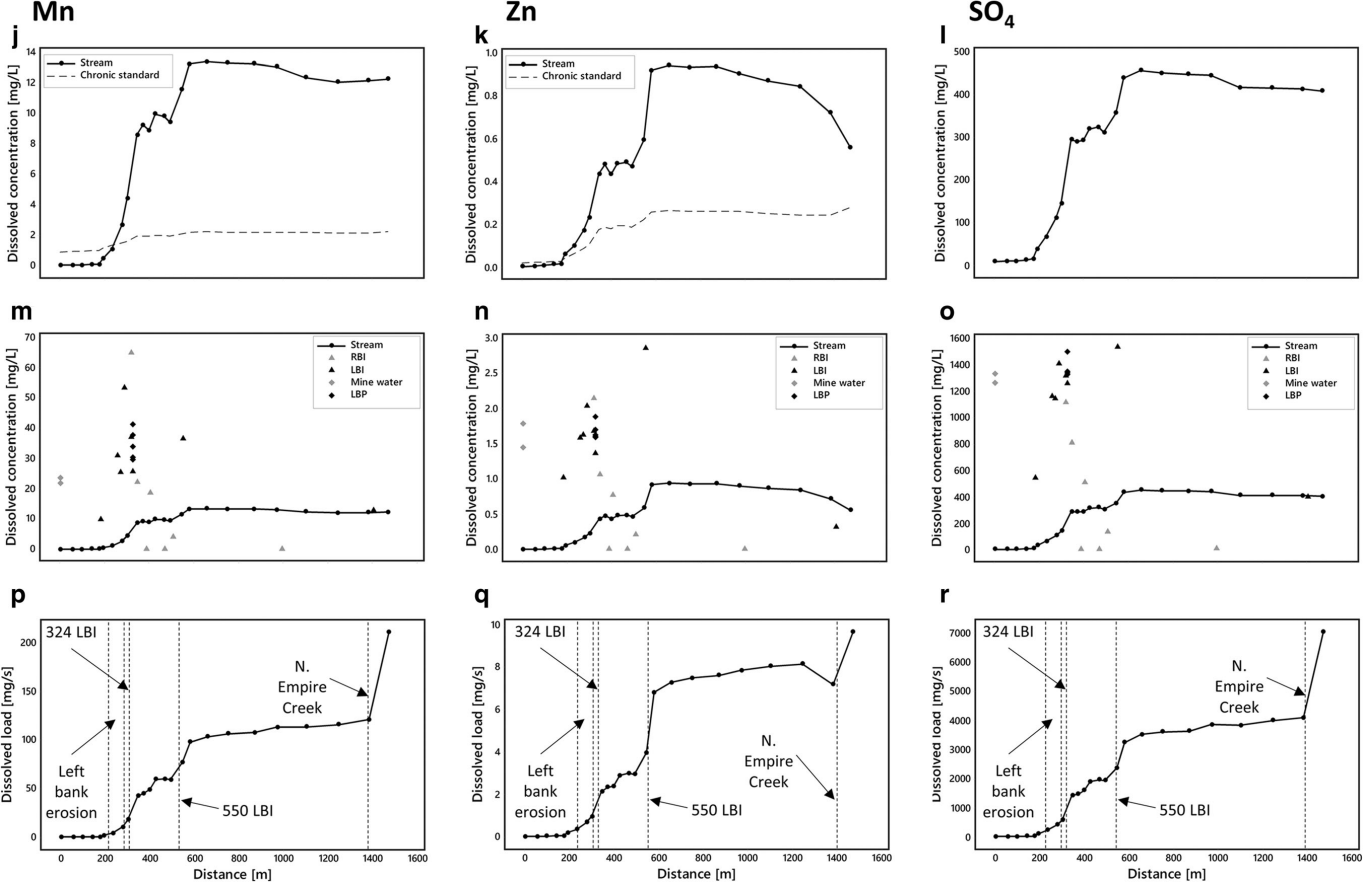

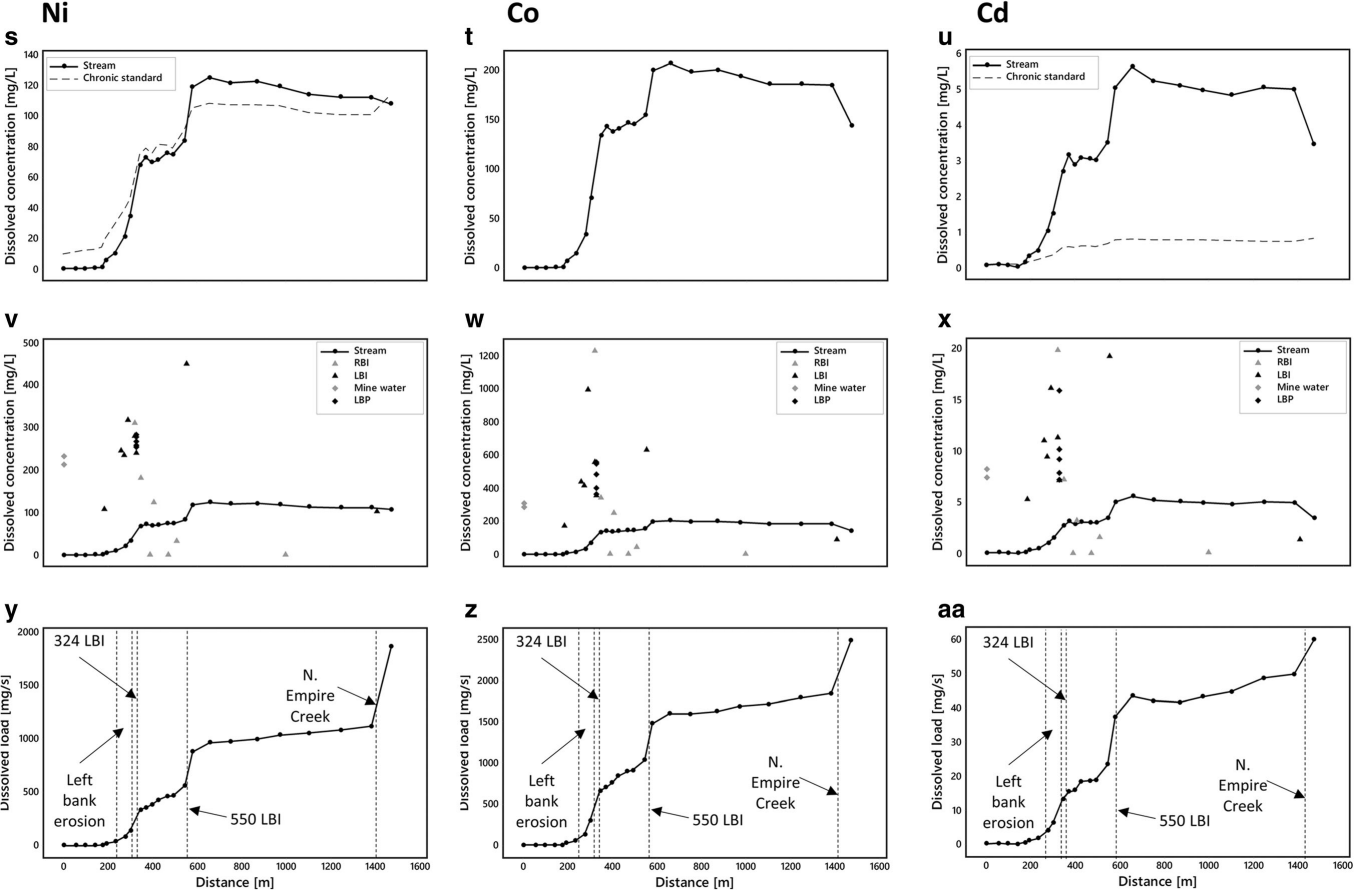
**a**–**c**, **j**–**l**, **s**–**u** Spatial profiles of dissolved stream constituents. **d**–**f**, **m**–**o**, **z**–**x** Spatial profiles of stream, inflow (*RBI* = right bank inflow; *LBI* = left bank inflow), mine water and piezometer (*LBP* = left bank piezometer) concentrations. **g**–**i**, **p**–**r**, **y**–**aa** Spatial profiles of constituent loads

Instream dissolved Al, Cu, Fe, Mn, Zn, Ni, and Cd concentrations generally exceed chronic aquatic life standards (Colorado Department of Public Health and Environment 2005) over most of the reach (Fig. 5a–c, j–l, s–u). Metals falling below the standards are Ag, As, Cr, Pb, and U and are not shown in Fig. 5 (see Supplementary Information). Metals exceeding the standards are generally below guidelines above ∼200 m (Ni above 579 m) and then exhibit a marked two-step increase in concentrations between ∼200 and 350 m and between ∼500 and 600 m. Iron behaves slightly differently with only one notable increase in concentrations between ∼200 and 400 m. Concentrations decrease gradually for all constituents from ∼600 m to the end of the study reach where North Empire Creek appears to have a diluting effect on all constituents except Fe and Mn. The locations of concentration increases along Lion Creek are consistent with high concentrations in left and right bank inflows (Fig. 5d–f, m–o, v–aa).

### Constituent loads

**Fig. 6:**
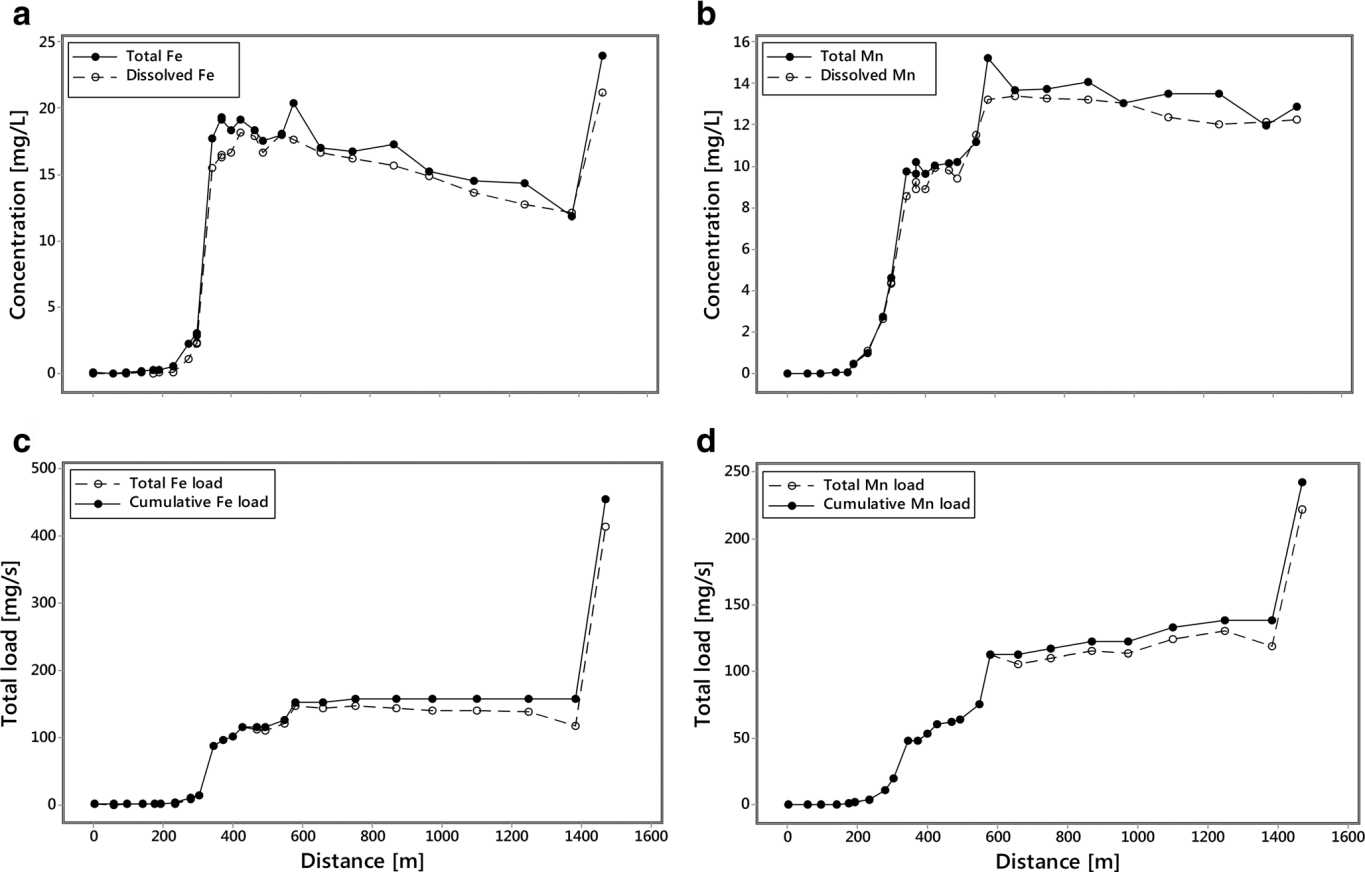
**a**, **b** Spatial profile of total recoverable and dissolved Fe and Mn concentrations. **c**, **d** Spatial profile of total and cumulative Fe and Mn loads

Changes in constituent loads obtained from synoptic sampling of watershed chemistry and tracer-derived discharge are illustrated in Fig. 3b and Fig. 5g–i, p–r, y–aa. Changes in load estimates for individual stream segments are shown in Table 3. Spatial loading profiles for the hydrogen ion show a sharp increase between ∼200 and 600 m followed by a steady addition of acidity to the end of the study reach. The other constituents demonstrate a different pattern with two sharp increases in loads in response to left bank inflows at 324 and 550 m followed by a steady increase thereafter. A notable exception is Fe which shows some loss of mass between ∼600 and 1400 m. Although dissolved loads of constituents generally equal total recoverable loads along the study reach suggesting conservative transport, Fe and Mn demonstrate reactive behaviour from ∼370 m coincident with the plateau in stream pH at ∼3.1 (Fig. 6a, b). The difference between the total instream load and the cumulative instream load can be used to quantify the amount of attenuation that occurs along the study reach. Attenuation of constituents is generally less than 1% suggesting limited natural attenuation via adsorption and precipitation processes. However, both Fe and Mn demonstrate greater attenuation downstream from ∼370 m coincident with the plateau in stream pH. Overall, attenuation of Fe is 5 and 8.8% for dissolved and total loads, respectively, and attenuation of Mn is 0.4 and 8.1% for dissolved and total loads, respectively (Fig. 6c, d).

**Fig. 7:**
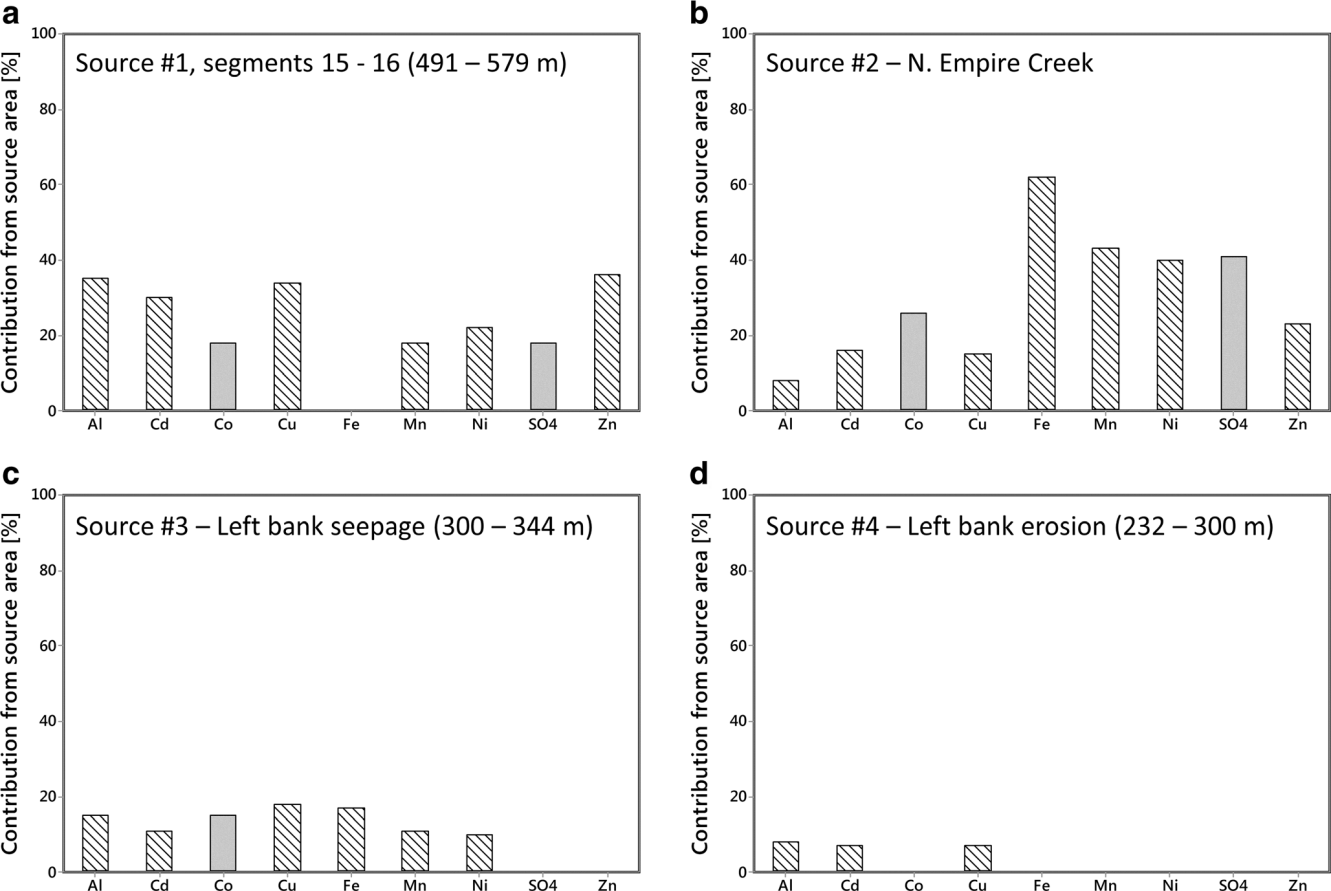
Percent contribution of source areas to overall load within the Lion Creek study reach. Percent contributions are based on dissolved concentrations. Constituents with concentrations in excess of chronic aquatic life standards (Colorado Department of Public Health and Environment 2005) are shown with *cross-hatched bars*

Calculation of the percent contribution of constituents within each stream segment allows for identification of the largest sources of individual constituents to the study reach. When one or more adjacent segments showed an increase in load, the segments were grouped to reflect loading from a general source area (Table 4). Four source areas account for most of the loading to Lion Creek. Stream segments 15 and 16 (491–579 m, Table 3; Source #1, Table 4) constitute the largest source area, exhibiting the largest load increases for Al, Cd, Cu, and Zn, and the second largest load increases for Mn, Ni, and SO_4_ (Fig. 7a). This source area includes a right bank inflow MN-0507 and left bank inflow MN-0550. The chemistry of the left bank inflow at 550 m highlights it as one of the main contributors of contaminants to Lion Creek which may have its source at the Minnesota mine shaft. However, comparison of the left bank inflow chemistry with the chemistry of the mine water samples (MN-ADIT, MN-POOL) suggests that few of the constituent concentrations (11 out of 28) are similar. North Empire Creek (stream segment 24, Table 3; Source #2, Table 4) is the second largest source, representing the largest single source of Fe, Mn, Ni, and SO_4_, and the second largest source of Cd and Zn (Fig. 7b). The third largest source area is stream segment 9 (300–344 m, Table 3; Source #3, Table 4), exhibiting the second largest load increases for Al, Cu, and Fe (Fig. 7c). This stream segment includes two left bank inflows from the seepage face (MN-0317, MN-0324) and right bank inflow MN-0318. Effective inflow concentrations in this segment are much closer to the seepage face inflows than they are to the right bank inflow suggesting the change in load through this segment is predominantly due to the seepage face inflows on the left bank, even though the right bank inflow has high constituent concentrations. Comparison of constituent concentrations observed in mine waters with the left bank inflow (including well samples) concentrations reveals that 19 out of 28 constituents are similar. The fourth identified source area is the left bank eroding area (232–300 m) that includes the left bank inflows at 258, 270, and 286 m (Fig. 7d).

**Table 4:** Percent loading of constituents of concern attributed to the four main source areas

	Source #1, segments 15–16 (491–579 m)	Source #2 N. Empire Creek	Source #3 Left Bank Seepage Face (300–344 m)	Source #4 Left Bank Erosion (232–300 m)
Al	*35*	8	15	8
Cd	*30*	16	11	7
Co	18	*26*	15	–
Cu	*34*	15	18	7
Fe	11	*62*	17	–
Mn	18	*43*	11	–
Ni	22	*40*	10	–
SO_4_	18	*41*	12	–
Zn	*36*	23	11	–

Only contributions >5% considered. Highest contributions in italics

### Principal components analysis

**Fig. 8:**
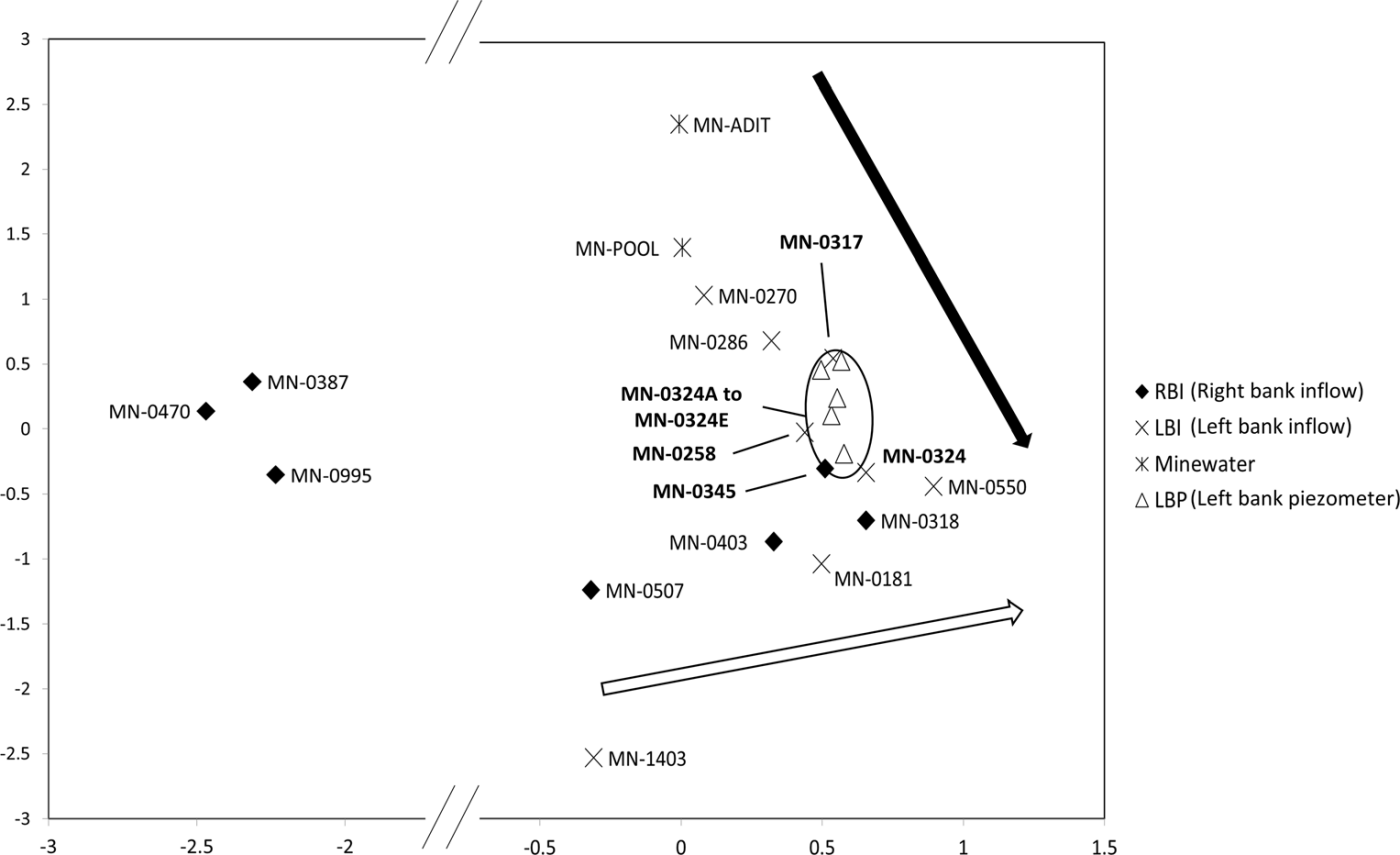
Biplot of sample loadings and variable scores for PCA of inflow, mine water, and well chemistry in the Lion Creek study reach. *Black and white arrows* represent the direction of the downstream increase in contaminant concentrations in left and right bank samples, respectively. The *x*-axis has been ‘broken’ in order to help visualise the associations between samples. *RBI* = right bank inflow; *LBI* = left bank inflow; *Mine water* = water samples taken at MN-ADIT and MN-POOL; *LBP* = left bank piezometer. [PC1 is the *x*-axis; PC2 is the *y*-axis]

Principal components analysis was conducted to help identify spatial patterns in constituent concentrations and to support source identification by synoptic sampling. Several assumption tests were performed on the data before analysis. The Kaiser-Meyer-Olkin (KMO) value was 0.86 indicating very good sampling adequacy and the Bartletts test of sphericity was highly significant (*p* < 0.001) indicating there was enough relationship between variables to perform the PCA. Two principal components were extracted from the analysis. A primary principal component (PC1—*x*-axis) explains 76% of the data variance and is characterised by the major contaminants including Al, Cd, Cu, Co, Cr, Fe, Mn, Ni, Pb, Zn, SO_4_, and pH. A second minor principal component (PC2—*y*-axis) explains a further 5% of the data variance and is characterised by V and As. Considering PC1, the contamination gradient, three right bank inflows (MN-0387, MN-0470, MN-0995) representing background water chemistry with low contaminant concentrations plot to the left of the biplot (Fig. 8). Samples plotting farther to the right have increasing contaminant concentrations. Of the contaminated inflows that originate from the left bank, contaminant concentrations increase in the downstream direction and also become less similar to the mine water samples. Samples from the eroding left bank area (MN-0270, MN-0286) plot closest to the mine water samples (MN-ADIT, MN-POOL), followed by samples from the seepage face area (MN-0317, MN-0324) and then MN-0550. A similar downstream increase in contaminant concentrations is observed in the right bank inflows.

## Discussion

### Constituent loads and implications for remediation

Examination of the spatial pattern of constituent loads in Lion Creek suggests four important source areas of contamination under low flow conditions (Fig. 7, Table 4). Two of these can be considered primary sources though the relative importance of individual constituents is different for each. Source area 1 (stream segments 15–16, 491–579 m, Table 4), including left bank inflow MN-0550, is the largest source of Al, Cd, Cu, and Zn (35, 30, 34, and 36%, respectively) and the second largest source of Mn, Ni, and SO_4_ (18, 22, and 18%, respectively). Source area 2, North Empire Creek, is the largest source of Fe, Mn, Ni, and SO_4_ (62, 43, 40, and 41%, respectively) and the second largest source of Cd and Zn (16 and 23%, respectively). All of these constituents (except SO_4_) fail to meet chronic aquatic life standards along most of the study reach. Source areas 3 (left bank seepage face, 300–344 m) and 4 (eroded area, 232–300 m) are responsible for less than 20 and 10% of constituent loading, respectively. These results suggest that remediation activities that are focused on source area 1 (MN-550 and vicinity) and North Empire Creek may have the greatest overall benefit under low flow conditions. Whilst the data presented in this study can be used to help prioritise remediation in Lion Creek, a separate synoptic sampling study and loading analysis would need to be undertaken in North Empire Creek upstream from the Lion Creek confluence in order to identify the primary sources of constituent loading in that watershed.

Source area 1 (491–597 m) accounts for >30% of Al, Cd, Cu, and Zn loading and ∼20% of Mn, Ni, and SO_4_ loading (six out of the seven constituents that exceed aquatic standards). Effective inflow concentrations suggest the primary source of contamination within this zone is the left bank inflow at MN-0550. On the day of synoptic sampling, this inflow consisted of two small surface inflows from a denuded area. A range of small-scale passive remediation options could be considered here to neutralise pH and remove dissolved metals (Byrne et al. 2012). For example, a vertical flow reactor (VLR) (Florence et al. 2016) that utilises topographical gradients and reactive media to remove metals and acidity from the contaminated inflow is one possible solution. However, thorough consideration of the desired effect of treatment as well as investigation of the suitability of different remediation options would be required before implementation of any treatment system. The effect of treating this inflow via remediation can be estimated through mass balance calculations. In the case of dissolved Cu, a post-remediation load near the end of the study reach (MN-1381) can be calculated by subtracting the load attributed to source area 1 from the pre-remediation load (7.62–2.55 = 5.07 mg/s). Dividing this by the flow at the end of the study reach (9.95 L/s) gives a post-remediation concentration of 0.51 mg/L, a reduction of 33%. The same calculation for Al, Cd, Fe, Mn, SO_4_, and Zn results in reductions of 27, 28, 21, 17, 21, and 40%, respectively. However, this intervention would not result in concentrations at MN-1381 falling below aquatic life standards. In addition, this calculation assumes that any remediation at MN-0550 removes 100% of the selected dissolved metals from the inflow, which is unlikely, and that it does not modify the flow attributed to that zone. This calculation also assumes that metal mass is not being removed from solution pre- or post-remediation by geochemical reactions. If dissolved metals are being removed by precipitation or adsorption reactions, then this calculation will likely overestimate constituent removal. The left bank inflow at MN-0550 is also a substantial source of acidity to Lion Creek. Remediation of this inflow that results in an increase in pH will modify precipitation and adsorption reactions most likely resulting in greater removal of dissolved Cu from the inflow. In this scenario, the calculation above may lead to underestimates of constituent removal. Due to the many assumptions and unknowns surrounding remediation, additional investigations (such as reactive transport modelling) would be necessary that account for pre-mining water quality and how remediation will change mass loading and instream geochemistry (Runkel and Kimball 2002; Runkel et al. 2007; Runkel 2010; Runkel et al. 2012).

After source area 1 and North Empire Creek, the next major source of constituent loading is the seepage face at MN-0324. Electrical resistivity imaging of the streambed in this area suggests that drainage from the seepage face may also be entering the stream via subsurface (hyporheic) pathways that link the stream bank to the streambed sediments (Johnston et al. 2017). Comparison of constituent concentrations between Minnesota Mine shaft and the seepage face suggests a hydraulic connection. In addition, PCA suggests a degree of connection between the Minnesota Mine shaft and left bank inflows at MN-0270, MN-0286, and MN-0550. Therefore, it could be argued that blocking this connection or treatment of the mine water at the source underground may have a greater overall benefit than attempting to treat the source of contamination in the river channel, as suggested above for MN-0550. A number of remediation options are available depending on the nature of the leakage. If the groundwater plume is diffuse in nature, an alternative to the conventional pump-and-treat approach would be the use of permeable reactive barriers (PRBs) (Byrne et al. 2012). PRBs have emerged in the last two decades as a cost-effective method for the treatment of diffuse groundwater contamination. Essentially, a PRB consists of an engineered trench in the pathway of a contamination plume that is backfilled with reactive material (typically zero valent iron and compost). Satisfactory neutralisation and dissolved constituent removal (including Al, Cd, Cu, Fe, Mn, Ni, SO_4_, and Zn) has been reported at PRB installations worldwide (Benner et al. 2002; Ludwig et al. 2009; Caraballo et al. 2010). The feasibility of utilising PRB technology at Minnesota Mine would need to be explored through additional investigations including direct physical examination of the underground mine workings and indirect geophysical techniques.

### Effect of rainfall runoff on constituent concentrations and loads

**Fig. 9:**
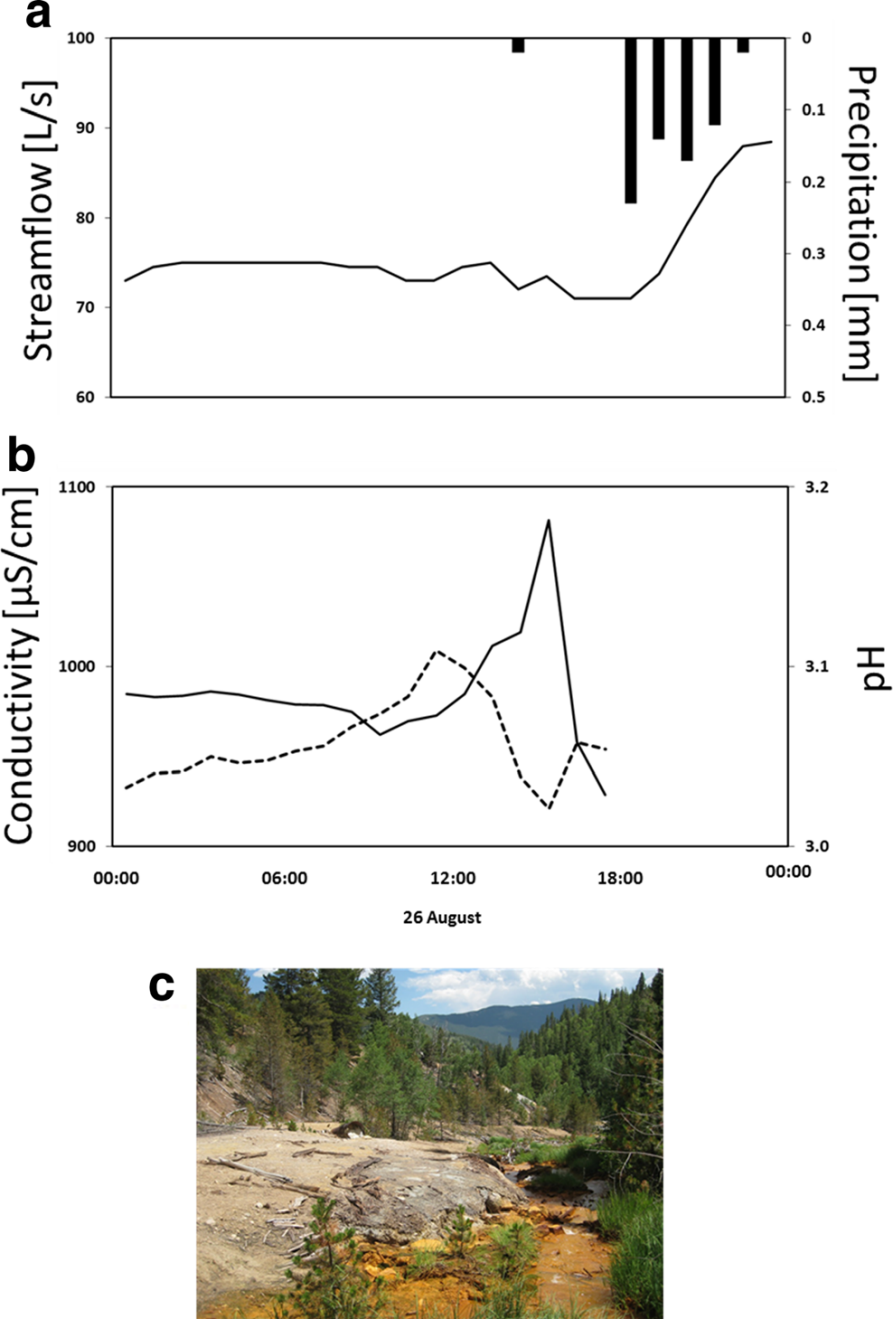
**a** Streamflow (recorded at USGS gage 06715000 on Clear Creek, approximately 3.5 km from study site; 10.5066/F7P55KJN) and precipitation (recorded at Berthound Pass, CO, approximately 8.5 km from study site (http://www.wrcc.dri.edu/cgi-bin/cliMAIN.pl?cobert)) for the period 25 to 27 August 2014. **b** Stream specific conductivity (*solid line*) and pH (*dashed line*) recorded at MN-0466 for the period 25 to 26 August 2014. **c** Image of eroding streamside tailings located between MN-0370 and MN-0398 along the left bank of the study reach

It is important to note that the loading results reported here are based on low flow conditions in Lion Creek in August 2014. These are valuable data as constituent concentrations in mining-affected watercourses are generally near their maximum in low flow conditions due to reduced dilution (Byrne et al. 2012). Low flows are therefore a critical period for the transport of dissolved constituents. In the present study, precipitation on site (witnessed by the authors) at the end of synoptic sampling on the 26 August (Fig. 9, nearest precipitation gage located 8.5 km from study site) caused stream pH to drop and specific conductivity to increase (pH and conductivity were recorded on-site at MN-0466), potentially due to dissolution of efflorescent salts in streamside tailings that released metals and acidity (Fig. 9). The stream gage in Fig. 9 is located 3.5 km downstream from Lion Creek on Clear Creek which explains the lag in flow change. A longer dataset for Lion Creek (June to October 2015) confirms the association of increased specific conductivity with rainfall-runoff events (Johnston et al. 2017). High river flows associated with rainfall-runoff events can substantially increase constituent concentrations and loads (Canovas et al. 2008; Banks and Palumbo-Roe 2010; Gozzard et al. 2011; Byrne et al. 2013; Nordstrom et al. 2015; Runkel et al. 2016) in streams with extensive streamside tailings (e.g. MN-0370 to MN-0398). This is because overland flow and rising stream water levels can connect source areas to the river channel that remain disconnected during dry conditions (for example, tailings located above the low flow water level). Typically, the greatest loads are associated with the first few hours of rainfall, a phenomenon known as the ‘first flush’ (Nordstrom 2011). This is mostly an issue in arid climates where prolonged oxidation of surface tailings produces efflorescent minerals that solubilise easily during rainfall, however flushing of metals and acidity has also been noted in temperate climates (Gozzard et al. 2011; Byrne et al. 2013). It is probable that substantial rainfall affecting the Lion Creek watershed will modify the spatial pattern of constituent concentrations and loads reported in this study, especially when this rainfall follows prolonged dry periods. For example, left bank tailings between 370 and 400 m have not been identified as a major source of metals and acidity in this study. This zone contributes little to overall streamflow (approximately 6%) and most of the streamside tailings were located above the stream water level during synoptic sampling. However, this zone is potentially a larger source of constituent loading during other (wetter) times of year, as are the other identified source areas. Remediation of the site in the 1990s (Holm 2012) that was aimed at improving water quality revegetated much of the tailings near the mine adit and introduced storm water controls (log revetments and rip-rap) in an eroding gully just downstream from MN-0095. However, several tailings deposits are still distributed throughout the study reach that are likely to become important sources of contamination during rainfall-runoff events.

### Source identification using synoptic sampling and principal components analysis

The synoptic sampling and mass balance approach for investigating mine drainage sources and processes was developed as part of the U.S. Geological Survey’s Toxic Substances Hydrology Program (Bencala et al. 1987; Kimball et al. 2002). The approach typically uses a constant-rate tracer injection to accurately determine streamflow and synoptic sampling to provide a detailed snapshot of constituent concentrations and loads (Runkel et al. 2013). Whilst use of a tracer may not always be possible (for example due to financial constraints or high river flows), streamflow and loading estimates based on tracer dilution should be seen as a prerequisite for studies aimed at gathering data to inform remediation activities. Streamflow estimates based on the traditional velocity-area method or even the more recent acoustic Doppler velocity method may introduce major uncertainties into loading and source area calculations due to error associated with irregular channels, non-logarithmic velocity profiles, and hyporheic flow (Jarrett 1984; Runkel et al. 2013). Constant-rate tracer injection also allows more sample sites to be measured over a short time period (no laborious flow measurements) allowing for more spatially dense and therefore more detailed estimates of streamflow and constituent loads. In addition, both flow and constituent concentrations are calculated from the same sample minimising any possible effect of temporal variation in stream chemistry or flow.

Principal components analysis can further develop the conceptual understanding of contaminant sources obtained from synoptic sampling especially when the sources of contamination may be diffuse and it is difficult to untangle their respective chemistries. The real benefit of PCA lies in the ability to identify patterns within the multi-variate synoptic data that are otherwise difficult to characterise and visualise. Variations in stream inflow trace metal chemistry as a result of mining and mineralization or as a result of different geological features can be revealed as distinct chemical signatures (Kimball et al. 2002; De Giudici et al. 2014). It is the ability of PCA to identify patterns in trace metal and major ion chemistry that makes this technique a more useful companion to synoptic sampling than more traditional geochemical techniques such as piper diagrams that only consider major ion chemistry. Analysis of inflow chemistry in Lion Creek clearly distinguishes between mine-affected waters and the background chemical signal of unaffected waters. More importantly, left and right bank inflows are shown to have distinct chemical signatures. Both are indicative of mining contamination but the particular chemistry of the left bank inflows suggests a hydraulic connection with the Minnesota Mine shaft. From this, we can hypothesise that a leakage in the Minnesota Mine shaft has created a plume of mine drainage that is following the topographical gradient and emerging as seeps and inflows along the left bank of Lion Creek. This possibility must be considered and further investigated prior to any remediation works in Lion Creek. Despite the clear advantages of using PCA in conjunction with tracer-derived synoptic data, relatively few studies (Kimball et al. 2002; De Giudici et al. 2014) have adopted this technique to help distinguish different sources of contamination.

## Conclusions

Application of a constant-rate tracer injection and synoptic sampling in the Lion Creek study reach suggests concentrations of Al, Cd, Cu, Fe, Mn, Ni, and Zn exceed chronic aquatic life standards. Constituent loading within the study reach is diffuse in nature with four primary sources identified along the left bank. Listed according to decreasing importance at low streamflow, these are source area 1 (491–579 m), North Empire Creek (1403 m), the left bank seepage face (300–344 m), and the left bank eroding area (232–300 m). Multi-variate analysis (PCA) of stream inflow chemistry suggests a hydraulic link between left bank inflows and mine water in the Minnesota Mine shaft. Together, synoptic sampling and PCA suggest constituent loading from North Empire Creek, streamside tailings in Lion Creek, and underground leakage of contaminated mine water from Minnesota Mine are the primary factors limiting water quality in the study reach.

Previous remediation of the Minnesota Mine site focussed on stabilisation of the large tailings deposits near the mine and an eroding gully that focussed storm water during high rainfall. However, many tailings deposits remain in the watershed and there is strong evidence for contaminated water from Minnesota Mine entering Lion Creek via subsurface pathways. As a result, remediation to improve water quality to acceptable standards may require a multi-method approach. The greatest benefit for low flow concentrations and loads may be achieved by preventing contaminated water from Minnesota Mine from entering Lion Creek. Stabilisation or removal of remaining streamside tailings may be an effective measure to reduce the potential for contamination episodes during rainfall-runoff events. Additional investigations focussed on establishing the location of potential leakage of the Minnesota Mine workings and also constituent loading in North Empire Creek are needed prior to the implementation of remedial actions.

## Electronic supplementary material

ESM 1 (PDF 598 kb).
