# Supplementary material for: Synoptic sampling and principal components analysis to identify sources of water and metals to an acid mine drainage stream
Source: Environ Sci Pollut Res Int. 2017 Jun 6;24(20):17220–40. doi: 10.1007/s11356-017-9038-x (PMC5508047; doi:10.1007/s11356-017-9038-x)
Supplement: Supplementary file 1 — (PDF 598 kb). [file 11356_2017_9038_MOESM1_ESM.pdf]

Supplementary Information for:

Synoptic sampling and principal components analysis to identify sources of water and metals to an acid mine drainage stream

Patrick Byrne[1,2], Robert L. Runkel[3], and Katherine Walton-Day[4]

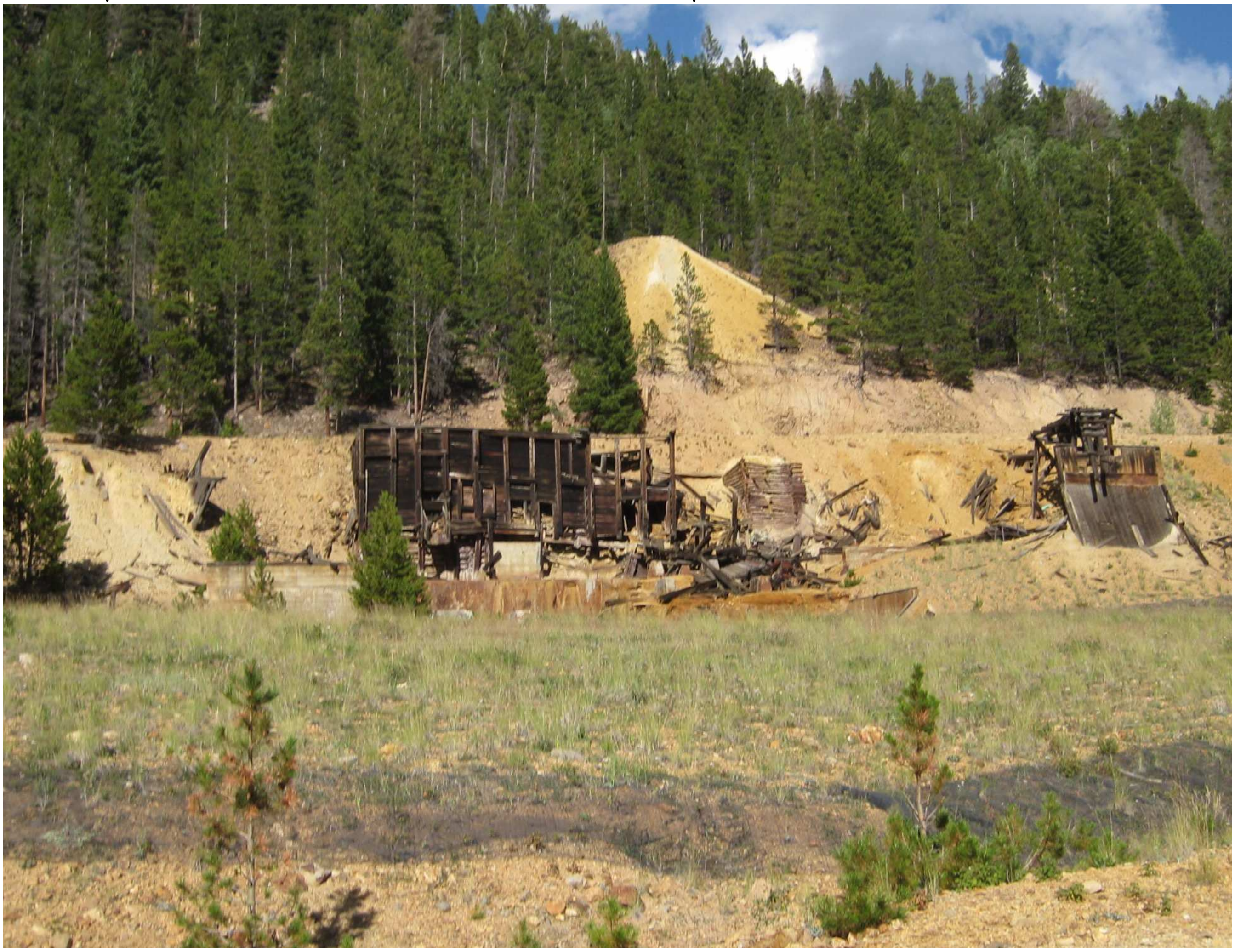

Environmental Science and Pollution Research; DOI:

Supplementary Information, 1 April 2017

1. School of Natural Sciences and Psychology, Liverpool John Moores University, Liverpool, L3 3AF, UK
2. Corresponding author: P.A.Byrne@ljmu.ac.uk
3. U.S. Geological Survey, 3215 Marine Street, Suite E127, Boulder, CO U.S.A., 80305
4. U.S. Geological Survey, Mail Stop 415, Denver Federal Center, Denver, CO U.S.A., 80225

Table S1. Sites sampled on August 26, 2014, Lion Creek, Colorado

[Source, type of sample collected where STR denotes stream sample, LBI denotes left bank inflow, and RBI denotes right bank inflow; Easting and Northing, Universal Transverse Mercator (UTM) coordinates in zone 13 S using the North American Datum of 1983 (NAD83)]

Site	Distance [meters]	Source	Description	Easting	Northing
MN-0000	0	STR	Lion Creek at injection site	440875	4403561
MN-0057	57	STR	Lion Creek at small waterfall, downstream from flume (flume installed 18 Oct. 2014)	440879	4403515
MN-0095	95	STR	Lion Creek, ~25 m upstream from dry, left bank drainage channel w/ riprap	440878	4403480
MN-0139	139	STR	Lion Creek upstream from left-bank outcrop (Transport site #1)	440867	4403441
MN-0174	174	STR	Lion Creek upstream from mossy inflow area	440868	4403409
MN-0181	181	LBI	Small depression in moss in area w/ multiple inflows	440872	4403404
MN-0191	191	STR	Lion Creek downstream from mossy inflow area	440875	4403394
MN-0232	232	STR	Lion Creek at downstream transect for electrical resistivity	440886	4403358
MN-0258	258	LBI	Two seeps along eroded area on left bank	440888	4403335
MN-0270	270	LBI	Seep along eroded area on left bank	440890	4403323
MN-0276	276	STR	Lion Creek within eroded left bank subreach	440892	4403318
MN-0286	286	LBI	Seep draining moss-covered, spongy soil at downstream end of eroded area	440893	4403308
MN-0300	300	STR	Lion Creek between eroded area and seepage face	440897	4403295
MN-0317	317	LBI	Inflow at upstream end of seepage face	440909	4403282
MN-0318	318	RBI	Right bank inflow w/ Ulothrix algae, at upstream end of left bank seepage face	440909	4403282
MN-0324	324	LBI	Left bank inflow along seepage face	440911	4403277
MN-0324	324	LBI	Piezometer nest installed in Left Bank seepage face; 3/8" stainless steel drive points w/ inlets at 10, 20, 30, 40, and 50 cm below ground surface (MN-0324A-E, respectively)	440911	4403277
MN-0344	344	STR	Lion Creek downstream from seepage face	440914	4403258
MN-0345	345	RBI	Right bank inflow in grass	440915	4403255
MN-0370	370	STR	Lion Creek upstream from left bank tailings	440919	4403233
MN-0387	387	RBI	Right bank inflow in grass/small bushes	440920	4403217
MN-0398	398	STR	Lion Creek upstream from split in stream	440928	4403209
MN-0403	403	RBI	Right bank inflow w/ Ulothrix algae	440932	4403206
MN-0425	425	STR	Lion Creek downstream from split in stream	440950	4403194
MN-0466	466	STR	Lion Creek upstream from dry, left bank drainage channel (Transport Site #2)	440955	4403154
MN-0470	470	RBI	Right bank inflow directly across from dry, left bank drainage channel	440954	4403150
MN-0491	491	STR	Lion Creek downstream from dry, left bank drainage channel	440967	4403136
MN-0507	507	RBI	Right bank inflow from grassy area	440980	4403127
MN-0545	545	STR	Lion Creek upstream from denuded area on left bank	441012	4403112
MN-0550	550	LBI	Two small surface inflows from denuded area on left bank	441017	4403113
MN-0579	579	STR	Lion Creek downstream from denuded area on left bank	441039	4403101
MN-0656	656	STR	Lion Creek upstream from left bank mine dump	441102	4403064
MN-0750	750	STR	Lion Creek in area with steep streambanks	441174	4403024
MN-0868	868	STR	Lion Creek upstream from left bank mine dump	441201	4402921
MN-0969	969	STR	Lion Creek in relatively flat area	441202	4402828
MN-0995	995	RBI	Right bank inflow from grassy area	441198	4402803
MN-1100	1100	STR	Lion Creek at switchback in road; upstream end of culvert	441240	4402712
MN-1245	1245	STR	Lion Creek along road in shady area	441299	4402582
MN-1381	1381	STR	Lion Creek at old wooden dam upstream from confluence w/ N. Empire Creek (TSite #3)	441355	4402479
MN-1403	1403	LBI	North Empire Creek near confluence with Lion Creek	441373	4402472
MN-1469	1469	STR	Downstream from Lion Creek/N Empire Creek confluence	441379	4402409
MN-ADIT			Adit of Minnesota Mine near shaft	441000	4403510
MN-POOL			Pooled water in shaft next to adit	441000	4403510

Table S2. Data for samples collected August 26, 2014, including tracer-dilution streamflow estimate, pH, specific conductance (SC), temperature, alkalinity, ^{2}H, and ^{18}O, Lion Creek, Colorado.

[Sample, "Site" from Table S1 with an optional letter suffix. "B" suffix for sites MN-0300 and MN-0370 denotes samples that are part of a field replicate; "A" –"E" suffixes for site MN-0324 denote multiple samples from nested piezometer]

Sample	Time	Streamflow [L/s]	pH	SC [µS/cm]	Temp. [°C]	Alkalinity [mg/L $CaCO_3$]	$\delta\,^{2}H$ [per mil]	$\delta\,^{18}O$ [per mil]
Stream Samples								
MN-0000	13:20	2.91	6.85	54.1	5.5	11.6	-136	-18.1
MN-0057	13:11	2.91	6.76	335	6	11.2	—	—
MN-0095	13:04	3.04	6.69	323	5.5	11.2	—	—
MN-0139	13:00	3.15	6.70	318	6	9.23	—	—
MN-0174	12:57	3.23	6.69	313	6	7.98	—	—
MN-0191	12:50	3.37	5.10	339	7	—	—	—
MN-0232	12:48	3.64	4.62	384	7	—	—	—
MN-0276	12:36	3.91	3.92	482	9	—	—	—
MN-0300	12:26	4.11	3.68	569	9.5	—	—	—
MN-0300B	12:28	4.11	3.69	567	9.5	—	—	—
MN-0344	12:10	4.90	3.09	971	9	—	—	—
MN-0370	12:00	4.97	3.09	999	10	—	—	—
MN-0370B	12:02	4.97	3.09	996	10	—	—	—
MN-0398	11:50	5.49	3.11	965	9.5	—	—	—
MN-0425	11:43	5.97	3.17	983	9.5	—	—	—
MN-0466	11:38	6.10	3.14	1004	10.5	—	—	—
MN-0491	11:20	6.25	3.15	969	9.5	—	—	—
MN-0545	11:33	6.68	3.14	1033	10	—	—	—
MN-0579	11:00	7.40	3.06	1214	14	—	—	—
MN-0656	10:53	7.72	3.15	1224	13.5	—	—	—
MN-0750	10:45	8.03	3.15	1218	12	—	—	—
MN-0868	10:37	8.13	3.14	1212	11.5	—	—	—
MN-0969	10:27	8.68	3.14	1198	9.5	—	—	—
MN-1100	10:12	9.22	3.08	1164	7	—	—	—
MN-1245	10:05	9.64	3.16	1134	7	—	—	—
MN-1381	09:52	9.95	3.08	1135	7	—	-132	-17.5
MN-1469	09:42	17.26	3.27	1044	7	—	—	—

Table S2. Data for samples collected August 26, 2014, including tracer-dilution streamflow estimate, pH, specific conductance (SC), temperature, alkalinity, ^{2}H, and ^{18}O, Lion Creek, Colorado. (Continued)

[Sample, "Site" from Table S1 with an optional letter suffix. "B" suffix for sites MN-0300 and MN-0370 denotes samples that are part of a field replicate; "A" –"E" suffixes for site MN-0324 denote multiple samples from nested piezometer]

Sample	Time	Streamflow [L/s]	pH	SC [µS/cm]	Temp. [°C]	Alkalinity [mg/L $CaCO_3$]	δ ^{2}H [per mil]	δ ^{18}O [per mil]
Inflow Samples								
MN-ADIT	13:36	—	2.74	2660	6	—	-140	-18.5
MN-POOL	13:40	—	2.88	2420	—	—	-139	-18.4
MN-0181	12:58	—	3.06	1250	9	—	-129	-17.1
MN-0258	12:43	—	2.68	2460	13	—	-131	-17.3
MN-0270	12:40	—	2.63	2530	14	—	-129	-17.1
MN-0286	12:33	—	2.76	2710	13	—	-129	-16.9
MN-0317	12:24	—	2.60	2830	13	—	-137	-18.2
MN-0318	12:17	—	2.84	2260	10	—	—	—
MN-0324	12:13	—	2.55	2820	11.5	—	—	—
MN-0324A	14:42	—	2.52	2970	—	—	—	—
MN-0324B	14:32	—	2.57	2770	—	—	—	—
MN-0324C	14:52	—	2.62	2870	—	—	—	—
MN-0324D	15:00	—	2.67	2880	—	—	—	—
MN-0324E	14:22	—	2.69	2840	—	—	—	—
MN-0345	12:06	—	2.65	2110	9.5	—	—	—
MN-0387	11:53	—	6.67	38.3	6	10.8	—	—
MN-0403	11:48	—	2.93	1393	11	—	-133	-17.6
MN-0470	11:30	—	6.88	50.2	7.5	15.9	—	—
MN-0507	11:17	—	3.67	360	12	—	-127	-16.7
MN-0550	11:08	—	2.66	2870	13	—	-135	-17.8
MN-0995	10:25	—	6.52	60.1	7	15.0	—	—
MN-1403	09:48	—	4.26	928	7	—	—	—
Blanks								
MN-BLANK	11:22							
MN-BLANK2	14:15							

Table S3. Total-recoverable concentrations from unfiltered samples collected August 26, 2014, Lion Creek, Colorado (Ag-Li)

[Total-recoverable concentrations for silver (Ag), aluminum (Al), arsenic (As), barium (Ba), calcium (Ca), cadmium (Cd), cobalt (Co), chromium (Cr), copper (Cu), iron (Fe), potassium (K), and lithium (Li), with concentration units of nanograms per liter (ng/L), micrograms per liter (µg/L), and milligrams per liter (mg/L) as specified below]

Sample	Ag (ng/L)	Al (mg/L)	As (ng/L)	Ba (µg/L)	Ca (mg/L)	Cd (µg/L)	Co (µg/L)	Cr (µg/L)	Cu (mg/L)	Fe (mg/L)	K (mg/L)	Li (mg/L)
Stream Sites												
MN-0000	<10	0.0492	46.2	10.9	4.93	0.0355	<0.1	0.0712	0.000371	0.0984	0.522	<0.008
MN-0057	<10	0.0232	73.1	14.3	5.33	<0.01	<0.1	0.0561	0.000656	0.0398	0.555	15.0
MN-0095	<10	0.0529	74.7	17.5	6.62	0.142	<0.1	0.0561	0.000635	0.127	0.645	13.9
MN-0139	<10	0.111	115	15.7	6.08	0.0982	0.632	0.0907	0.0125	0.168	0.552	13.6
MN-0174	<10	0.242	141	15.7	7.50	0.0700	1.12	0.0827	0.0155	0.221	0.706	12.9
MN-0191	<10	1.87	131	15.5	9.47	0.314	7.58	0.148	0.0672	0.302	0.740	12.0
MN-0232	<10	2.81	106	15.3	11.8	0.481	14.9	0.236	0.0969	0.509	0.663	11.6
MN-0276	<10	5.41	73.3	16.5	18.8	1.13	34.6	0.561	0.152	2.26	0.775	10.5
MN-0300	<10	7.59	66.4	15.1	22.7	1.54	71.2	0.744	0.235	3.04	0.851	10.2
MN-0300B	<10	7.57	87.1	14.4	22.9	1.56	70.2	0.726	0.233	2.86	0.860	10.2
MN-0344	10.3	16.5	77.5	13.0	44.0	2.80	137	1.68	0.528	17.7	0.870	8.46
MN-0370	13.0	16.2	99.3	15.5	43.5	3.17	146	1.72	0.554	19.3	0.883	8.57
MN-0370B	12.2	16.8	80.3	14.0	44.1	2.82	145	1.72	0.556	19.2	0.877	8.58
MN-0398	10.9	15.5	105	13.3	40.8	2.42	140	1.63	0.498	18.3	0.883	7.60
MN-0425	<10	15.7	70.7	12.9	41.4	3.06	144	1.73	0.487	19.2	0.853	7.09
MN-0466	12.2	16.2	118	12.7	42.7	3.09	152	1.63	0.522	18.4	0.810	7.02
MN-0491	11.9	16.6	97.9	13.1	43.0	2.99	141	1.61	0.479	17.5	0.853	6.54
MN-0545	18.1	17.8	93.3	14.0	47.1	3.67	158	2.00	0.556	18.0	0.814	6.25
MN-0579	17.0	29.5	80.6	13.2	62.6	5.46	208	3.90	0.873	19.8	0.983	5.67

Table S3. Total-recoverable concentrations from unfiltered samples collected August 26, 2014, Lion Creek, Colorado (Ag-Li) (Continued)

[Total-recoverable concentrations for silver (Ag), aluminum (Al), arsenic (As), barium (Ba), calcium (Ca), cadmium (Cd), cobalt (Co), chromium (Cr), copper (Cu), iron (Fe), potassium (K), and lithium (Li), with concentration units of nanograms per liter (ng/L), micrograms per liter (µg/L), and milligrams per liter (mg/L) as specified below]

Sample	Ag (ng/L)	Al (mg/L)	As (ng/L)	Ba (µg/L)	Ca (mg/L)	Cd (µg/L)	Co (µg/L)	Cr (µg/L)	Cu (mg/L)	Fe (mg/L)	K (mg/L)	Li (mg/L)
MN-0656	19.3	27.3	86.4	11.9	56.3	5.34	203	2.97	0.861	18.5	0.901	5.36
MN-0750	19.1	26.1	56.6	11.8	56.1	5.20	205	3.09	0.870	18.4	0.928	5.36
MN-0868	18.1	28.1	58.9	11.7	58.3	5.14	203	3.21	0.853	17.5	0.936	5.17
MN-0969	21.8	25.9	97.0	12.2	54.8	5.53	197	2.80	0.829	16.1	0.965	4.88
MN-1100	21.6	25.6	88.4	12.8	53.5	4.92	187	2.81	0.788	15.2	0.961	4.50
MN-1245	20.8	26.0	50.3	12.7	54.3	4.84	184	2.93	0.766	14.3	1.01	4.25
MN-1381	25.1	23.8	56.2	13.7	50.6	4.57	187	2.58	0.787	11.8	0.958	4.19
MN-1469	22.1	15.4	319	22.2	62.5	3.83	148	1.78	0.537	24.0	2.82	2.46
Inflows												
MN-0470	19.4	0.134	112	26.2	4.31	0.0205	0.654	0.126	0.00366	0.484	0.593	0.00826
MN-0995	40.3	0.388	105	34.4	5.02	<0.01	0.423	0.447	0.00695	0.400	0.497	0.0146
MN-1403	13.6	1.78	618	33.6	84.9	1.31	94.8	0.272	0.175	48.8	5.72	0.0187
Blanks and Detection Limit												
MN-BLANK	<10	<0.007	<20	<0.02	<0.037	<0.01	<0.1	0.0130	<0.0001	<0.005	<0.013	<0.008
MN-BLANK2	<10	0.0116	<20	<0.02	<0.037	0.0379	<0.1	0.0282	<0.0001	0.0136	<0.013	0.0103
Det. Lim.	10	0.007	20	0.02	0.037	0.01	0.1	0.002	0.0001	0.005	0.013	0.008

Table S4. Total-recoverable concentrations from unfiltered samples collected August 26, 2014, Lion Creek, Colorado (Mg-Zn)

[Total-recoverable concentrations for and magnesium (Mg), manganese (Mn), molybdenum (Mo), sodium (Na), nickel (Ni), lead (Pb), silicon (Si), sulfate (SO_4), strontium (Sr), uranium (U), vanadium (V), and zinc (Zn), with concentration units of micrograms per liter (µg/L), and milligrams per liter (mg/L) as specified below]

Sample	Mg (mg/L)	Mn (mg/L)	Mo (µg/L)	Na (mg/L)	Ni (µg/L)	Pb (µg/L)	Si (mg/L)	SO_4[a] (mg/L)	Sr (µg/L)	U (µg/L)	V (µg/L)	Zn (mg/L)
Stream Sites												
MN-0000	1.03	0.0137	0.191	3.30	0.393	0.0631	7.68	11.4	42.9	0.228	0.100	0.00602
MN-0057	1.11	0.00529	0.404	11.0	0.402	0.173	7.11	10.8	47.1	0.148	0.0865	0.00692
MN-0095	1.38	0.0180	0.420	11.9	0.519	0.140	8.08	12.3	62.2	0.145	0.102	0.0117
MN-0139	1.26	0.0389	0.350	9.58	0.880	0.193	7.07	13.1	55.3	0.373	0.0730	0.0136
MN-0174	1.61	0.0820	0.333	10.5	1.51	0.244	7.95	18.0	60.0	0.447	0.0833	0.0209
MN-0191	2.71	0.472	0.299	9.66	6.35	0.217	7.94	40.4	71.5	4.03	0.0632	0.0691
MN-0232	3.64	0.963	0.235	8.75	10.5	0.287	8.11	58.3	82.1	7.28	0.0542	0.0938
MN-0276	6.27	2.72	0.220	9.78	21.6	0.403	10.8	113	111	11.4	0.133	0.177
MN-0300	7.85	4.60	0.177	9.57	34.7	0.326	12.0	150	136	15.8	0.161	0.246
MN-0300B	7.76	4.59	0.173	9.32	34.3	0.317	11.8	145	127	15.7	0.156	0.257
MN-0344	17.1	9.76	0.150	10.8	67.8	0.337	18.6	332	179	30.5	0.198	0.498
MN-0370	16.6	9.64	0.169	10.2	72.7	0.688	18.3	322	174	32.9	0.309	0.504
MN-0370B	17.2	10.2	0.140	10.4	73.0	0.462	19.2	342	169	32.6	0.246	0.519
MN-0398	16.3	9.66	0.197	9.38	70.5	0.333	16.9	318	170	29.0	0.278	0.485
MN-0425	16.3	10.0	0.242	8.94	72.9	0.305	16.5	317	180	29.6	0.352	0.491
MN-0466	16.4	10.2	0.217	8.77	77.2	0.431	16.7	315	176	31.6	0.301	0.525
MN-0491	16.6	10.2	0.275	8.92	73.5	0.367	17.6	319	170	30.7	0.271	0.529
MN-0545	17.6	11.2	0.348	8.60	85.7	0.753	18.6	344	184	35.8	0.371	0.579
MN-0579	27.0	15.2	0.331	10.2	124	0.353	23.6	489	228	56.0	0.305	0.922

Table S4. Total-recoverable concentrations from unfiltered samples collected August 26, 2014, Lion Creek, Colorado (Mg-Zn) (Continued)

[Total-recoverable concentrations for and magnesium (Mg), manganese (Mn), molybdenum (Mo), sodium (Na), nickel (Ni), lead (Pb), silicon (Si), sulfate (SO_4), strontium (Sr), uranium (U), vanadium (V), and zinc (Zn), with concentration units of micrograms per liter (µg/L), and milligrams per liter (mg/L) as specified below]

Sample	Mg (mg/L)	Mn (mg/L)	Mo (µg/L)	Na (mg/L)	Ni (µg/L)	Pb (µg/L)	Si (mg/L)	SO_4[a] (mg/L)	Sr (µg/L)	U (µg/L)	V (µg/L)	Zn (mg/L)
MN-0656	24.0	13.7	0.232	8.80	123	0.378	21.0	436	200	59.4	0.209	0.940
MN-0750	23.8	13.7	0.212	8.57	124	0.367	20.4	424	193	58.9	0.203	0.942
MN-0868	25.0	14.1	0.201	9.23	123	0.405	21.7	468	207	58.0	0.181	0.929
MN-0969	23.2	13.1	0.168	8.45	119	0.370	20.6	424	199	56.1	0.150	0.895
MN-1100	23.3	13.5	0.210	8.53	115	0.336	20.6	436	195	53.8	0.131	0.868
MN-1245	23.4	13.5	0.209	8.70	111	0.370	21.1	430	198	52.0	0.128	0.858
MN-1381	21.3	11.9	0.176	7.81	114	0.521	19.4	380	197	52.5	0.106	0.735
MN-1469	25.7	12.9	0.131	8.63	110	1.50	17.2	395	254	31.3	0.202	0.601
Inflows												
MN-0470	1.19	0.0615	2.43	3.14	0.484	0.436	7.78	5.72	32.4	0.369	0.301	0.00275
MN-0995	1.41	0.0203	4.40	3.52	0.352	1.03	8.67	9.03	45.4	0.451	0.611	0.00526
MN-1403	33.1	14.6	0.0707	10.6	107	2.45	14.6	459	329	2.70	0.0421	0.366
Blanks and Detection Limit												
MN-BLANK	<0.022	<0.002	<0.005	<0.07	<0.06	0.00336	<0.036	<0.162	<0.2	<0.03	<0.006	<0.001
MN-BLANK2	<0.022	0.00557	<0.005	<0.07	<0.06	0.00599	0.0365	<0.162	<0.2	<0.03	<0.006	<0.001
Det. Lim.	0.022	0.002	0.005	0.07	0.06	0.002	0.036	0.162	0.2	0.03	0.006	0.001

a. SO_4 concentrations were calculated using the sulfur (S) results obtained by ICP-MS, under the assumption that all S was in the form of SO_4.

Table S5. Dissolved concentrations from filtered samples collected August 26, 2014, Lion Creek, Colorado (Ag-Li)

[Dissolved concentrations for silver (Ag), aluminum (Al), arsenic (As), barium (Ba), calcium (Ca), cadmium (Cd), cobalt (Co), chromium (Cr), copper (Cu), iron (Fe), potassium (K), and lithium (Li), with concentration units of nanograms per liter (ng/L), micrograms per liter (µg/L), and milligrams per liter (mg/L) as specified below]

Sample	Ag (ng/L)	Al (mg/L)	As (ng/L)	Ba (µg/L)	Ca (mg/L)	Cd (µg/L)	Co (µg/L)	Cr (µg/L)	Cu (mg/L)	Fe (mg/L)	K (mg/L)	Li (mg/L)
Stream Sites												
MN-0000	<10	0.0127	26.5	9.9	4.28	0.103	<0.1	0.0411	0.000428	0.0148	0.446	<0.008
MN-0057	<10	<0.007	152	14.8	5.03	0.122	<0.1	0.0400	0.000333	0.0099	0.547	14.7
MN-0095	<10	<0.007	42.0	15.8	5.49	0.102	<0.1	0.0473	0.000672	0.0113	0.584	13.8
MN-0139	<10	0.0794	65.7	15.7	5.87	0.0463	0.558	0.0720	0.00899	0.123	0.600	13.3
MN-0174	<10	0.0274	100	16.1	6.35	0.175	1.05	0.0344	0.00758	0.0158	0.596	12.4
MN-0191	36.1	0.776	24.7	15.9	8.93	0.353	7.22	0.0427	0.0630	0.0451	0.676	12.0
MN-0232	<10	2.58	94.5	15.8	13.4	0.502	14.3	0.112	0.0897	0.129	0.756	11.3
MN-0276	<10	5.08	52.3	15.1	18.8	1.05	33.8	0.394	0.148	1.10	0.798	10.4
MN-0300	<10	7.68	38.1	15.1	21.9	1.55	71.2	0.668	0.231	2.27	0.836	10.2
MN-0300B	<10	7.52	25.7	15.7	21.5	1.53	71.9	0.649	0.237	2.27	0.809	10.1
MN-0344	<10	14.4	58.3	12.7	37.5	2.72	134	1.67	0.519	15.5	0.778	8.41
MN-0370	<10	14.7	96.0	12.7	40.1	3.18	143	1.85	0.541	16.5	0.854	8.40
MN-0370B	<10	15.0	22.5	12.7	38.8	3.06	140	1.63	0.530	16.3	0.787	8.20
MN-0398	<10	14.3	43.1	13.5	37.9	2.90	138	1.53	0.492	16.6	0.793	7.51
MN-0425	<10	15.5	87.3	13.1	41.1	3.09	141	1.59	0.483	18.2	0.863	6.91
MN-0466	<10	16.3	122	12.4	41.3	3.06	147	1.68	0.501	17.9	0.813	6.76
MN-0491	<10	15.7	38.5	13.1	40.1	3.03	145	1.61	0.492	16.6	0.809	6.60
MN-0545	10.5	18.7	43.9	12.7	47.9	3.52	155	1.84	0.539	18.1	0.882	6.17
MN-0579	13.5	25.8	53.7	12.6	52.9	5.06	200	3.02	0.831	19.7	0.832	5.57

Table S5. Dissolved concentrations from filtered samples collected August 26, 2014, Lion Creek, Colorado (Ag-Li) (Continued)

[Dissolved concentrations for silver (Ag), aluminum (Al), arsenic (As), barium (Ba), calcium (Ca), cadmium (Cd), cobalt (Co), chromium (Cr), copper (Cu), iron (Fe), potassium (K), and lithium (Li), with concentration units of nanograms per liter (ng/L), micrograms per liter (µg/L), and milligrams per liter (mg/L) as specified below]

Sample	Ag (ng/L)	Al (mg/L)	As (ng/L)	Ba (µg/L)	Ca (mg/L)	Cd (µg/L)	Co (µg/L)	Cr (µg/L)	Cu (mg/L)	Fe (mg/L)	K (mg/L)	Li (mg/L)
MN-0656	14.2	27.1	100	11.6	54.3	5.64	207	2.99	0.873	18.5	0.892	5.34
MN-0750	15.3	26.8	76.5	12.2	54.1	5.25	199	3.12	0.843	17.8	0.867	5.14
MN-0868	12.5	26.9	81.8	11.7	53.7	5.12	200	2.82	0.846	17.5	0.889	5.07
MN-0969	16.4	26.4	64.9	11.7	53.8	4.99	194	2.90	0.816	16.2	0.931	4.75
MN-1100	16.8	24.9	38.9	12.9	51.0	4.85	186	2.76	0.780	15.0	0.905	4.47
MN-1245	18.2	24.5	71.0	13.3	50.4	5.06	186	2.55	0.784	14.1	0.952	4.28
MN-1381	20.7	24.5	61.6	14.1	50.5	5.01	185	2.74	0.766	12.1	0.977	4.14
MN-1469	<10	15.3	272	21.4	59.0	3.48	144	1.66	0.519	21.2	2.63	2.39
Inflows												
MN-ADIT	21.0	27.4	140	0.830	120	8.28	309	13.0	5.48	332	0.765	0.0473
MN-POOL	16.0	23.7	275	7.17	122	7.44	283	10.5	4.54	314	1.31	0.0397
MN-0181	22.6	44.8	23.2	1.85	59.3	5.29	170	2.12	1.20	3.54	1.66	0.0451
MN-0258	18.9	64.8	123	4.14	121	11.0	436	8.08	1.63	73.2	0.708	0.0820
MN-0270	21.0	72.5	386	3.79	96.6	9.46	413	10.5	2.10	68.2	0.0431	0.0814
MN-0286	33.0	103	261	3.97	151	16.1	994	5.00	2.78	36.1	1.85	0.103
MN-0317	13.4	55.7	40.5	0.448	130	11.3	556	7.62	2.28	134	0.352	0.0628
MN-0318	34.8	73.3	109	4.61	128	19.8	1229	5.78	2.66	19.9	1.54	0.292
MN-0324	18.9	49.9	32.9	0.869	128	7.14	353	7.25	2.37	105	0.128	0.0560
MN-0324A	15.0	58.7	59.7	0.855	146	7.22	364	10.1	2.32	131	0.221	0.0587
MN-0324B	14.5	56.9	60.4	1.49	138	7.84	398	10.1	2.29	147	0.303	0.0710
MN-0324C	19.0	58.7	59.5	1.66	133	9.21	484	14.1	2.22	186	0.507	0.0679
MN-0324D	16.3	68.2	140	2.40	147	15.9	557	44.6	2.34	228	0.697	0.0707

Table S5. Dissolved concentrations from filtered samples collected August 26, 2014, Lion Creek, Colorado (Ag-Li) (Continued)

[Dissolved concentrations for silver (Ag), aluminum (Al), arsenic (As), barium (Ba), calcium (Ca), cadmium (Cd), cobalt (Co), chromium (Cr), copper (Cu), iron (Fe), potassium (K), and lithium (Li), with concentration units of nanograms per liter (ng/L), micrograms per liter (μg/L), and milligrams per liter (mg/L) as specified below]

Sample	Ag (ng/L)	Al (mg/L)	As (ng/L)	Ba (μg/L)	Ca (mg/L)	Cd (μg/L)	Co (μg/L)	Cr (μg/L)	Cu (mg/L)	Fe (mg/L)	K (mg/L)	Li (mg/L)
MN-0324E	15.9	59.5	38.1	2.26	135	10.2	548	10.3	2.22	202	0.588	0.0672
MN-0345	13.2	36.0	37.3	6.68	83.1	7.18	340	7.97	1.51	50.2	1.18	0.0471
MN-0387	<10	0.0361	24.7	10.5	2.68	<0.01	0.153	0.0580	0.000488	0.0726	0.542	<0.008
MN-0403	<10	23.7	23.4	11.3	60.1	3.18	249	0.928	0.260	33.0	1.30	0.427
MN-0470	<10	0.0163	59.9	22.3	4.03	0.0130	<0.1	0.0551	0.00196	0.0515	0.573	<0.008
MN-0507	<10	5.14	47.2	33.3	19.7	1.60	40.8	0.389	0.111	3.66	0.993	0.0138
MN-0550	25.6	108	34.4	2.69	121	19.2	631	18.2	4.40	62.7	0.268	0.109
MN-0995	<10	0.0306	45.4	27.6	4.68	0.0546	<0.1	0.0547	0.00237	0.0228	0.436	0.0104
MN-1403	<10	1.52	460	34.3	73.6	1.36	88.8	0.163	0.162	37.1	5.19	0.0107
Blanks and Detection Limit												
MN-BLANK	<10	<0.007	<20	<0.02	<0.037	<0.01	<0.1	0.0147	<0.0001	<0.005	<0.013	<0.008
MN-BLANK2	<10	<0.007	<20	<0.02	<0.037	0.177	<0.1	0.0277	<0.0001	<0.005	<0.013	<0.008
Det. Lim.	10	0.007	20	0.02	0.037	0.01	0.1	0.002	0.0001	0.005	0.013	0.008

Table S6. Dissolved concentrations from filtered samples collected August 26, 2014, Lion Creek, Colorado (Mg-Zn)

[Dissolved concentrations for and magnesium (Mg), manganese (Mn), molybdenum (Mo), sodium (Na), nickel (Ni), lead (Pb), silicon (Si), sulfate (SO_4), strontium (Sr), uranium (U), vanadium (V), and zinc (Zn), with concentration units of micrograms per liter (μg/L), and milligrams per liter (mg/L) as specified below]

Sample	Mg (mg/L)	Mn (mg/L)	Mo (μg/L)	Na (mg/L)	Ni (μg/L)	Pb (μg/L)	Si (mg/L)	SO_4[a] (mg/L)	Sr (μg/L)	U (μg/L)	V (μg/L)	Zn (mg/L)
Stream Sites												
MN-0000	0.903	0.00310	0.190	2.83	0.412	0.00793	6.57	9.76	41.5	0.138	0.0780	0.00541
MN-0057	1.06	0.00251	0.395	10.3	0.424	0.0298	6.87	10.2	46.6	0.108	0.0693	0.00668
MN-0095	1.16	0.00286	0.388	9.71	0.416	0.0252	6.82	10.1	51.5	0.122	0.0738	0.00966
MN-0139	1.29	0.0516	0.362	9.71	0.947	0.0621	7.01	13.3	54.0	0.263	0.0610	0.0156
MN-0174	1.43	0.0690	0.332	9.15	1.30	0.00421	6.91	15.6	58.4	0.238	0.0439	0.0182
MN-0191	2.59	0.441	0.229	9.32	6.06	0.112	7.67	37.8	69.2	3.14	0.0143	0.0617
MN-0232	4.14	1.06	0.0568	10.0	10.5	0.150	9.15	67.9	87.9	6.68	<0.006	0.102
MN-0276	6.22	2.64	0.0173	9.67	21.3	0.204	10.8	111	106	11.2	<0.006	0.175
MN-0300	7.71	4.39	0.0357	9.20	34.6	0.220	11.9	146	129	16.1	0.0101	0.234
MN-0300B	7.63	4.34	0.0453	9.01	34.0	0.241	11.8	143	131	15.9	0.0120	0.232
MN-0344	14.6	8.58	0.0979	9.05	68.2	0.245	16.2	295	164	30.1	0.146	0.438
MN-0370	15.6	9.22	0.111	9.47	73.0	0.431	16.0	290	168	31.4	0.181	0.482
MN-0370B	15.3	8.89	0.0956	9.14	69.9	0.309	16.7	306	169	30.4	0.152	0.458
MN-0398	14.7	8.88	0.161	8.56	69.9	0.305	16.0	293	172	28.6	0.225	0.436
MN-0425	16.5	9.94	0.193	9.03	71.4	0.280	16.9	320	182	28.9	0.288	0.485
MN-0466	16.1	9.80	0.166	8.49	76.0	0.321	17.2	324	167	30.8	0.255	0.491
MN-0491	15.6	9.43	0.245	8.26	74.8	0.340	16.8	312	168	30.4	0.235	0.472
MN-0545	18.4	11.5	0.216	8.98	84.0	0.335	18.3	357	179	35.6	0.194	0.595
MN-0579	23.9	13.2	0.235	8.41	119	0.352	20.7	439	203	56.1	0.203	0.921
MN-0656	25.0	13.4	0.202	8.47	125	0.315	21.4	457	195	58.3	0.170	0.943
MN-0750	24.5	13.3	0.190	8.33	122	0.302	20.9	451	202	55.5	0.164	0.934

Table S6. Dissolved concentrations from filtered samples collected August 26, 2014, Lion Creek, Colorado (Mg-Zn) (Continued)

[Dissolved concentrations for and magnesium (Mg), manganese (Mn), molybdenum (Mo), sodium (Na), nickel (Ni), lead (Pb), silicon (Si), sulfate (SO_4), strontium (Sr), uranium (U), vanadium (V), and zinc (Zn), with concentration units of micrograms per liter (μg/L), and milligrams per liter (mg/L) as specified below]

Sample	Mg (mg/L)	Mn (mg/L)	Mo (μg/L)	Na (mg/L)	Ni (μg/L)	Pb (μg/L)	Si (mg/L)	SO_4[a] (mg/L)	Sr (μg/L)	U (μg/L)	V (μg/L)	Zn (mg/L)
MN-0868	24.6	13.3	0.171	8.31	123	0.313	21.1	448	199	56.5	0.138	0.938
MN-0969	24.3	13.0	0.150	8.30	120	0.289	21.1	446	197	54.1	0.132	0.906
MN-1100	23.3	12.4	0.200	7.95	114	0.325	20.3	417	203	53.1	0.111	0.873
MN-1245	22.7	12.0	0.163	7.82	112	0.371	20.3	416	186	51.4	0.0900	0.846
MN-1381	22.6	12.2	0.165	7.78	112	0.458	20.3	413	203	51.1	0.0871	0.723
MN-1469	25.8	12.2	0.0576	8.20	108	1.14	17.1	408	246	30.9	0.0282	0.561
Inflows												
MN-ADIT	48.9	23.5	0.0786	6.86	232	0.0817	19.8	1331	254	104	13.8	1.78
MN-POOL	48.7	21.9	0.0752	6.45	213	0.587	18.1	1264	285	90.1	10.0	1.45
MN-0181	26.0	9.58	0.0104	7.45	107	0.142	24.2	546	336	96.7	0.0261	1.02
MN-0258	50.0	30.9	0.124	10.5	245	0.371	52.0	1159	482	106	0.369	1.58
MN-0270	48.0	25.4	0.208	9.77	235	0.210	52.9	1145	327	154	1.46	1.62
MN-0286	58.3	53.2	0.131	11.7	317	0.516	57.5	1409	613	204	3.15	2.03
MN-0317	61.3	36.9	0.0260	10.0	279	0.231	44.6	1317	372	110	1.18	1.68
MN-0318	49.3	64.6	0.0661	9.93	309	0.587	44.2	1115	531	143	0.157	2.14
MN-0324	57.9	25.4	0.0361	9.12	239	0.254	39.4	1255	353	126	0.156	1.37
MN-0324A	64.8	29.6	0.0472	11.0	254	0.337	44.9	1352	359	110	0.233	1.60
MN-0324B	61.2	30.3	0.0465	10.7	258	0.333	45.0	1339	371	109	0.474	1.59
MN-0324C	60.9	33.8	0.0714	10.8	267	0.472	44.0	1327	407	107	0.890	1.62
MN-0324D	68.9	41.1	0.0944	12.4	282	1.16	51.6	1497	408	113	2.11	1.88
MN-0324E	59.8	37.6	0.0409	10.9	278	0.259	46.3	1340	401	103	1.19	1.70
MN-0345	39.9	22.1	0.0400	7.66	180	1.34	31.3	808	266	82.2	0.940	1.06
MN-0387	0.716	<0.002	2.58	2.64	0.0834	0.0469	6.56	4.64	22.2	0.100	0.110	<0.001

Table S6. Dissolved concentrations from filtered samples collected August 26, 2014, Lion Creek, Colorado (Mg-Zn) (Continued)

[Dissolved concentrations for and magnesium (Mg), manganese (Mn), molybdenum (Mo), sodium (Na), nickel (Ni), lead (Pb), silicon (Si), sulfate (SO_4), strontium (Sr), uranium (U), vanadium (V), and zinc (Zn), with concentration units of micrograms per liter (µg/L), and milligrams per liter (mg/L) as specified below]

Sample	Mg (mg/L)	Mn (mg/L)	Mo (µg/L)	Na (mg/L)	Ni (µg/L)	Pb (µg/L)	Si (mg/L)	SO_4[a] (mg/L)	Sr (µg/L)	U (µg/L)	V (µg/L)	Zn (mg/L)
MN-0403	23.8	18.5	0.0274	6.05	124	0.360	21.7	512	216	23.4	0.139	0.764
MN-0470	1.15	0.00974	2.57	3.01	0.260	0.0462	7.09	5.51	31.6	0.138	0.126	0.00190
MN-0507	7.38	3.98	0.0267	4.66	31.4	0.614	10.6	136	75.3	8.62	0.0675	0.209
MN-0550	91.6	36.2	0.0692	10.1	450	0.322	49.4	1536	333	278	0.118	2.85
MN-0995	1.28	<0.002	4.57	3.19	0.295	0.0656	8.08	8.34	45.7	0.0458	0.0622	0.00427
MN-1403	30.6	12.6	0.0138	8.88	103	1.28	12.3	403	331	2.53	0.00664	0.316
Blanks and Detection Limit												
MN-BLANK	<0.022	<0.002	<0.005	<0.07	<0.06	0.0141	<0.036	<0.162	<0.2	0.0311	0.00690	<0.001
MN-BLANK2	<0.022	<0.002	<0.005	<0.07	<0.06	0.00463	0.0412	<0.162	<0.2	<0.03	<0.006	<0.001
Det. Lim.	0.022	0.002	0.005	0.07	0.06	0.002	0.036	0.162	0.2	0.03	0.006	0.001

a. SO_4 concentrations were calculated using the sulfur (S) results obtained by ICP-MS, under the assumption that all S was in the form of SO_4.

Table S7. Dissolved concentrations from filtered samples for bromide (Br), chloride (Cl), fluoride (F), ferrous iron [Fe(II)], and ferrous plus ferric iron [Fe(II+III)], Lion Creek, Colorado, August 26, 2014. Bromide and chloride concentrations in most stream samples are elevated due to the injection of lithium chloride and sodium bromide.

[Sample, "Site" from Table S1 with an optional letter suffix. "B" suffix for sites MN-0300 and MN-0370 denotes samples that are part of a field replicate; "A" –"E" suffixes for site MN-0324 denote multiple samples from nested piezometer]

Sample	Br (mg/L)	Cl (mg/L)	F (mg/L)	Fe(II) (mg/L)	Fe(II+III) (mg/L)
Stream Samples					
MN-0000	<0.03	0.21	0.30	0.016	0.016
MN-0057	29.3	76.9	0.21	0.008	0.010
MN-0095	28.0	73.2	0.27	0.011	0.011
MN-0139	27.0	70.3	0.25	0.036	0.125
MN-0174	26.4	68.0	0.25	0.038	0.110
MN-0191	25.3	64.1	0.39	0.043	0.046
MN-0232	23.4	59.1	0.78	0.105	0.123
MN-0276	21.8	54.9	0.92	0.445	1.14
MN-0300	20.7	52.2	0.99	1.10	2.58
MN-0300B	20.7	52.3	0.95	1.00	2.56
MN-0344	17.0	43.8	1.27	2.69	17.5
MN-0370	17.1	42.6	1.19	3.95	18.2
MN-0370B	17.1	43.1	1.22	3.88	18.3
MN-0398	15.6	39.5	1.28	5.54	19.0
MN-0425	14.5	36.5	1.28	7.72	20.5
MN-0466	14.0	35.5	1.20	9.24	20.5
MN-0491	13.4	33.8	1.30	9.25	18.6
MN-0545	12.7	32.0	1.21	6.02	19.0
MN-0579	11.2	29.3	0.90	5.39	20.6
MN-0656	10.7	27.9	0.91	4.58	18.9
MN-0750	10.6	27.7	0.91	4.15	18.8
MN-0868	10.4	27.0	0.88	3.66	17.9
MN-0969	9.71	25.3	0.94	2.47	16.4
MN-1100	9.21	23.3	0.91	2.07	16.0
MN-1245	8.80	21.8	1.01	1.51	14.4
MN-1381	8.32	21.1	0.93	1.23	13.7
MN-1469	4.90	14.1	1.08	15.8	24.3

Table S7. Dissolved concentrations from filtered samples for bromide (Br), chloride (Cl), fluoride (F), ferrous iron [Fe(II)], and ferrous plus ferric iron [Fe(II+III)], Lion Creek, Colorado, August 26, 2014. Bromide and chloride concentrations in most stream samples are elevated due to the injection of lithium chloride and sodium bromide. (Continued)

[Sample, "Site" from Table S1 with an optional letter suffix. "B" suffix for sites MN-0300 and MN-0370 denotes samples that are part of a field replicate; "A" –"E" suffixes for site MN-0324 denote multiple samples from nested piezometer]

Sample	Br (mg/L)	Cl (mg/L)	F (mg/L)	Fe(II) (mg/L)	Fe(II+III) (mg/L)
Inflow Samples					
MN-ADIT	<0.03	0.83	9.58	305	355
MN-POOL	<0.03	0.73	9.45	278	354
MN-0181	<0.03	0.93	3.93	0.268	4.10
MN-0258	<0.03	0.90	5.53	14.2	81.8
MN-0270	<0.03	1.23	4.59	1.52	75.3
MN-0286	<0.03	1.04	4.26	20.1	41.9
MN-0317	<0.03	1.13	7.48	84.9	152
MN-0318	0.61	0.81	7.36	1.26	22.7
MN-0324	<0.03	0.83	8.90	0.726	119
MN-0324A	<0.03	1.23	8.83	11.6	126
MN-0324B	<0.03	0.97	8.31	53.2	147
MN-0324C	<0.03	1.04	7.83	161	197
MN-0324D	<0.03	1.06	6.94	226	226
MN-0324E	<0.03	1.04	7.49	204	225
MN-0345	<0.03	0.81	6.55	11.7	57.5
MN-0387	<0.03	0.38	0.20	0.049	0.063
MN-0403	0.62	0.72	5.09	16.1	39.8
MN-0470	<0.03	0.38	0.23	0.055	0.055
MN-0507	<0.03	0.62	0.62	3.47	3.70
MN-0550	<0.03	1.90	6.32	1.21	70.5
MN-0995	<0.03	0.75	0.27	0.008	0.008
MN-1403	<0.03	4.75	0.87	40	42.2
Blanks					
MN-BLANK	<0.03	<0.05	<0.05	0.018	0.024
MN-BLANK2	<0.03	<0.05	<0.05	<0.002	0.002

Table S8. Relationship between Sample IDs used herein and IDs used in USGS National Water Information System (NWIS)

[Sample, "Site" from Table S1 with an optional letter suffix. "B" suffix for sites MN-0300 and MN-0370 denotes samples that are part of a field replicate; "A" –"E" suffixes for site MN-0324 denote multiple samples from nested piezometer]

NWIS Site Number (C1)	NWIS Station Name (C12)	NWIS Medium Code	Sample (Table S2)
394646105412001	MN-ADIT MINNESOTA MINE ADIT	WG	MN-ADIT
394646105412002	MN-POOL IN GRATED CHAMBER	WG	MN-POOL
394648105412601	MN-0000 LION CREEK INJECTION SITE	WS	MN-0000
394646105412501	MN-0057 LION CR 57 M DWNSTRM FR INJ SITE	WS	MN-0057
394646105412502	MN-0095 LION CR 95 M DWNSTRM FR INJ SITE	WS	MN-0095
394644105412601	MN-0139 LION CR 139 M DWNSTRM FR INJ-TRANS SITE1	WS	MN-0139
394643105412601	MN-0174 LION CR 174 M DWNSTRM FR INJ SITE	WS	MN-0174
394643105412602	MN-0181 LION CR LFT BNK INF AT 181M DS FR INJ SITE	WG	MN-0181
394642105412601	MN-0191 LION CR 191 M DWNSTRM FR INJ SITE	WS	MN-0191
394641105412501	MN-0232 LION CR 232 M DWNSTRM FR INJ SITE	WS	MN-0232
394640105412501	MN-0258 LION CR LFT BNK INF AT 258M DS FR INJ SITE	WG	MN-0258
394640105412502	MN-0270 LION CR LFT BNK INF AT 270M DS FR INJ SITE	WG	MN-0270
394640105412503	MN-0276 LION CR 276 M DWNSTRM FR INJ SITE	WS	MN-0276
394640105412504	MN-0286 LION CR LFT BNK INF AT 286M DS FR INJ SITE	WG	MN-0286
394639105412501	MN-0300 LION CR 300 M DWNSTRM FR INJ SITE	WS	MN-0300
394639105412501	MN-0300 LION CR 300 M DWNSTRM FR INJ SITE	WSQ	MN-0300B
394639105412402	MN-0317 LION CR LFT BNK INF AT 317M DS FR INJ SITE	WG	MN-0317
394639105412403	MN-0318 LION CR RT BNK INF AT 318M DS FR INJ SITE	WG	MN-0318
394639105412404	MN-0324 LION CR LFT BNK INF AT 324M DS FR INJ SITE	WG	MN-0324
394639105412405	MN-0324A LION CR PIEZO 10 CM DEEP AT 324 M DS INJ	WG	MN-0324A
394639105412406	MN-0324B LION CR PIEZO 20 CM DEEP AT 324 M DS INJ	WG	MN-0324B
394639105412407	MN-0324C LION CR PIEZO 30 CM DEEP AT 324 M DS INJ	WG	MN-0324C
394639105412408	MN-0324D LION CR PIEZO 40 CM DEEP AT 324 M DS INJ	WG	MN-0324D
394639105412409	MN-0324E LION CR PIEZO 50 CM DEEP AT 324 M DS INJ	WG	MN-0324E
394638105412401	MN-0344 LION CR 344 M DWNSTRM FR INJ SITE	WS	MN-0344
394638105412402	MN-0345 LION CR RTBNK INF AT 345M DS FR INJ SITE	WG	MN-0345
394637105412401	MN-0370 LION CR 370 M DWNSTRM FR INJ SITE	WS	MN-0370
394637105412401	MN-0370 LION CR 370 M DWNSTRM FR INJ SITE	WSQ	MN-0370B
394637105412402	MN-0387 LION CR RT BNK INF AT 387M DS FR INJ SITE	WG	MN-0387
394636105412301	MN-0398 LION CR 398 M DWNSTRM FR INJ SITE	WS	MN-0398

Table S8. Relationship between Sample IDs used herein and IDs used in USGS National Water Information System (NWIS)

[Sample, "Site" from Table S1 with an optional letter suffix. "B" suffix for sites MN-0300 and MN-0370 denotes samples that are part of a field replicate; "A" –"E" suffixes for site MN-0324 denote multiple samples from nested piezometer]

NWIS Site Number (C1)	NWIS Station Name (C12)	NWIS Medium Code	Sample (Table S2)
394636105412302	MN-0403 LION CR RTBNK INF AT 403M DS FR INJ SITE	WG	MN-0403
394636105412201	MN-0425 LION CR 425 M DWNSTRM FR INJ SITE	WS	MN-0425
394635105412201	MN-0466 LION CR 466 M DWNSTRM FR INJ-TRANS SITE 2	WS	MN-0466
394634105412201	MN-0470 LION CR RT BNK TRIB AT 470M DS FR INJ SIT	WS	MN-0470
394634105412202	MN-0491 LION CR 491 M DWNSTRM FR INJ SITE	WS	MN-0491
394634105412101	MN-0507 LION CR RT BNK INF AT 507M DS FR INJ SITE	WG	MN-0507
394633105412001	MN-0545 LION CR 545 M DWNSTRM FR INJ SITE	WS	MN-0545
394633105412002	MN-0550 LION CR LFT BNK INF AT 550 DS FR INJ SITE	WG	MN-0550
394633105411901	MN-0579 LION CR 579 M DWNSTRM FR INJ SITE	WS	MN-0579
394632105411601	MN-0656 LION CR 656 M DWNSTRM FR INJ SITE	WS	MN-0656
394630105411301	MN-0750 LION CR 750 M DWNSTRM FR INJ SITE	WS	MN-0750
394627105411201	MN-0868 LION CR 868 M DWNSTRM FR INJ SITE	WS	MN-0868
394624105411201	MN-0969 LION CR 969 M DWNSTRM FR INJ SITE	WS	MN-0969
394623105411201	MN-0995 LION CR RT BNK INF AT 995 DS FR INJ SITE	WG	MN-0995
394620105411001	MN-1100 LION C 1100 M DWNSTRM FR INJ SITE	WS	MN-1100
394616105410701	MN-1245 LION C 1245 M DWNSTRM FR INJ SITE	WS	MN-1245
394613105410501	MN-1381 LION C 1381 M DWNSTRM FR INJ-TRANS SITE 3	WS	MN-1381
394613105410401	MN-1403 EMPIRE C 1403 M DWNSTRM FR LION C INJ SITE	WS	MN-1403
394611105410401	MN-1469 LION C 1469 M DWNSTRM FR INJ SITE	WS	MN-1469
394634105412202	MN-0491 LION CR 491 M DWNSTRM FR INJ SITE	OAQ	MN-BLANK
394623105411201	MN-0995 LION CR RT BNK INF AT 995 DS FR INJ SITE	OAQ	MN-BLANK2

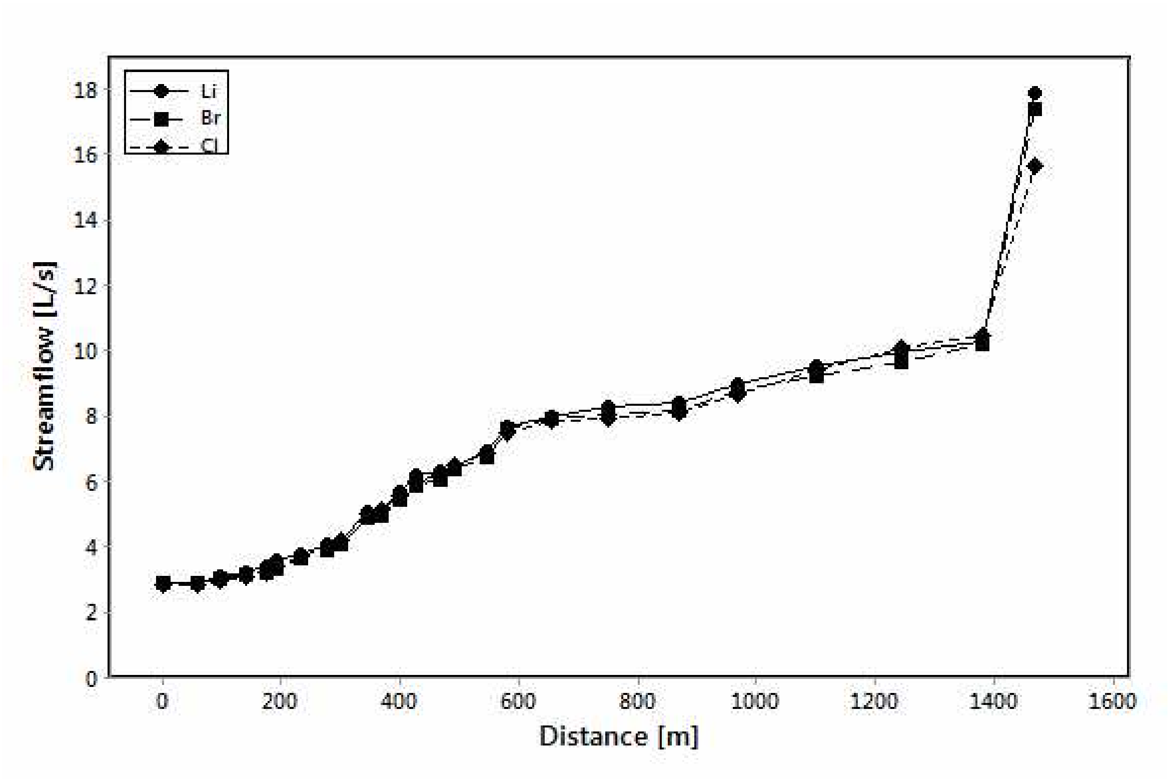

Figure S1. Tracer-dilution streamflow estimates based on observed dilution of Br, Cl, and Li.
